# The Therapeutic Impact of Plant-Based and Nutritional Supplements on Anxiety, Depressive Symptoms and Sleep Quality among Adults and Elderly: A Systematic Review of the Literature

**DOI:** 10.3390/ijerph20065171

**Published:** 2023-03-15

**Authors:** Darshan Kamat, Yazan A. Al-Ajlouni, Ryan C. W. Hall

**Affiliations:** 1 College of Medicine, University of Central Florida, Orlando, FL 32827, USA; 2 School of Medicine, New York Medical College, Valhalla, NY 10595, USA

**Keywords:** anxiety symptoms, depressive symptoms, sleep quality, supplements, randomized control trial

## Abstract

Background: The emerging research in the literature continues to forecast a drastic and alarming increase in negative mental health and sleep health outcomes among populations, especially after the COVID-19 pandemic, which significantly influenced people’s way of life. With mental health pharmaceutical interventions continuing to be stigmatized and inaccessible among populations, natural supplements provide an opportunity for intervention. Objective: This study sought to conduct a systematic review of the literature on the most recent comprehensive evidence for which nutritional supplements have the greatest therapeutic impact on symptoms of anxiety, depression, and insomnia. Methods: A systematic search of the literature, utilizing several databases, including PubMed and Web of Science, was conducted on 29 April 2022. We used developed keywords and MeSH terms for the search. The study eligibility criteria included (1) a randomized control trial; (2) investigating a plant-based therapeutic or natural supplement as the intervention; (3) measuring at least one health outcome of the following: anxiety symptoms, depressive symptoms, or sleep health outcomes; (4) utilizing validated measurement tools to measure the outcome of interest; (5) written in the English language; (6) peer reviewed; and (7) focused on adults and elderly populations. Main Results: Following the PRISMA guidelines, 76 studies were included in this review. We used the revised Risk of Bias tool (RoB2) to assess the quality of all included randomized control trials. A qualitative data synthesis was conducted. Overall, we found several valuable insights from the evidence in the literature, including evidence that demonstrates the benefits of probiotics and vitamin B complexes on anxiety symptoms, depressive symptoms, and sleep quality. Implication of Key Findings: This review provides the most updated findings in the literature on the topic, including an abundance of research that was published in the past 5 years. Given the expected rise in negative mental and sleep health outcomes following the pandemic, the supplements and therapeutics identified in this study should be the target of intervention measures to increase their accessibility and affordability and allow them to be incorporated into clinical guidelines of treatment. PROSPERO registration number: CRD42022361130.

## 1. Introduction

According to the National Health and Nutrition Examination Survey from the CDC in 2017–2018, 57.6% of U.S. adults used a dietary supplement in a 30-day survey period. The survey found that for all age categories, women used more dietary supplements than men, and with advancing age, the usage of several dietary supplements rose. Almost one-quarter of people aged 60 and up reported using four or more dietary supplements. Multivitamin–mineral supplements were the most frequent dietary supplements used by adults of all ages, followed by vitamin D and omega-3 fatty acid products [1]. In all age categories, the percentage of adults reporting dietary supplement use grew from 2007–2008 to 2017–2018. Many people in the United States take dietary supplements, which can help them receive the nutrients they need, but this also makes it more likely that they will take too many of them, especially if they use a lot of different supplements at the same time.

According to the World Health Organization (WHO), depression is a leading cause of disability worldwide, contributing significantly to the global disease burden [2]. Anxiety is another common condition that can co-occur with depression or other medical conditions. At some point in their lives, one out of every five people in the world meets the clinical criteria for an anxiety disorder, and this is frequently accompanied by a sleep disorder [3]. The American Academy of Sleep Medicine defines insomnia as difficulty falling or staying asleep, which causes daytime deficits pertaining to those sleeping problems. It is estimated that 9 to 15 percent of the world’s population suffers from insomnia, which has serious consequences during the day [4]. The interplay of depression, anxiety, and insomnia not only has an impact on people’s work and daily lives, but also has a negative impact on their quality of life and perceived well-being. Because of the concurrent increase in supplement use and the incidence of these psychiatric conditions, it is important to aggregate evidence for which of these interventions has any significant impact on symptom severity.

Emerging research in the literature has increasingly been focusing on the effects of such supplements on psychiatric disorders, including anxiety, depression, and sleep health. Several reviews of the clinical effectiveness of herbal and nutrient treatments for depression, anxiety disorders, and sleep disturbance have been published over the past decade [5,6,7,8,9]. Despite that, the mechanism in which the association between plant-based and natural supplements with anxiety, depressive, and sleep health symptoms occurs has not yet been fully established in the literature. Their uses previously have been linked to their adaptogenic, anti-stress, and anti-inflammatory properties [10].

Previous reviews have concentrated on a single mental health condition or a narrow range of interventions involving herbal, vitamin, and mineral supplementation. However, there has been an increased consumption of not only supplementary vitamins and minerals, amino acids, and pre- and probiotics, but also adaptogens from ayurveda and traditional Chinese medicine in the last 5–10 years, because of increased health supplement marketing and popularity [11]. Upon this basis, we sought to conduct a systematic review of the literature on the most recent comprehensive evidence for which nutritional supplements have the greatest therapeutic impact on symptoms of anxiety, depression, and insomnia. We also wanted to know which populations these supplements are safe for.

Many patients rely on cursory web searches or education from supplement marketing without a thorough understanding of the level of evidence supporting their claims. This review aims to serve as a jumping-off point for evidence-based discussions between providers and patients on appropriate and individualized treatment plans. Upon these bases, we sought to conduct a systematic review of the literature to summarize the available data in the literature on the effectiveness of plant-based and natural supplement intervention programs on anxiety, depressive, and sleep health symptoms among the adults and elderly populations globally. To the best of our knowledge, this is one of the first studies to have reviewed the evidence on the therapeutic impact of plant-based and nutritional supplements on anxiety, depressive, and sleep health symptoms all together. Additionally, this review is one of the latest reviews updating the evidence considering the projected increase in mental health outcomes following the COVID-19 pandemic.

## 2. Methods

### 2.1. Literature Search

A systematic literature search was performed in accordance with PRISMA (Preferred Reporting Items for Systematic Reviews and Meta-Analyses) guidelines [12]. On 29 April 2022, we conducted a systematic literature review on multiple databases, including PubMed, MEDLINE, Embase, CINAHL, and Web of Science. A set of specific keywords and MeSH terms was developed and is shown in supplementary Table S1. The protocol of this systematic review is registered with PROSPERO (international prospective register of systematic reviews) with the ID CRD42022361130

### 2.2. Search Strategy and Selection Criteria

**Figure 1:**
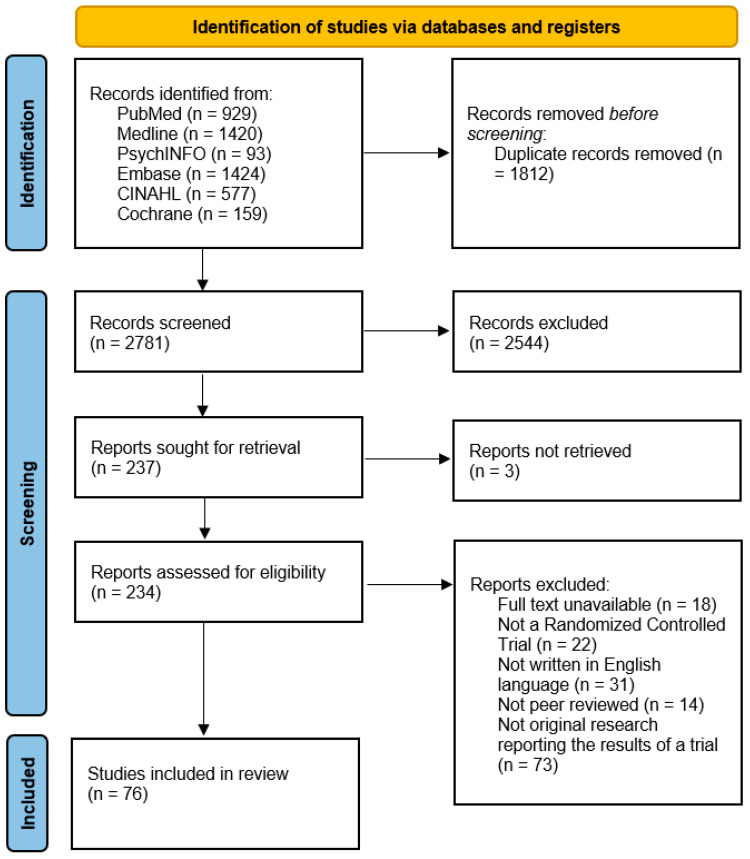
Preferred Reporting Items for Systematic Reviews and Meta-Analyses (PRISMA) study selection flow diagram outlining the literature review process when searching for articles on PubMed and Web of Science.

A set of keywords was developed by two authors. Then, it was reviewed and developed further by a third author to ensure its relevance to the research question. Upon utilizing the keywords to search the databases of choice, relevant articles were obtained and entered into Endnote, which is a referencing software. Using Endnote, duplicates were identified and removed. Upon removing duplicates, all articles of concern were entered into Rayyan, a free web-tool for screening articles for systematic reviews [13]. Two authors (Y.A.A.-A. and D.K.) then performed title and abstract screening concurrently, with conflicts resolved by a third author. As shown in Figure 1, full article screening in more depth was performed on all included studies from the previous stage of screening. Articles included until this stage were deemed eligible if (1) they were a randomized control trial; (2) they investigated a plant-based therapeutic or natural supplement as the intervention; (3) they measured at least one health outcome of the following: anxiety symptoms, depressive symptoms, or sleep health outcomes; (4) they utilized validated measurement tools to measure the outcome of interest; (5) they were written in English language; and (6) if they were peer reviewed. Similarly, articles were excluded if they were (1) not peer-reviewed; (2) written in a language that is not English; or (3) did not have full-text available publicly. Finally, where multiple unique papers were based on the same dataset, the paper with the biggest number of variables and largest dataset was included.

### 2.3. Definitions

#### 2.3.1. Plant-Based and Natural Supplement

The term “plant-based“ nutritional supplement can include dietary supplements that are said to help with various aspects of cognition and health. They are made of fruits, vegetables, nuts, seeds, spices, bark, flowers, leaves, and other botanical ingredients. The source of those supplements depends on the nutritional purposes of the supplements and can be made up of vitamins, minerals, herbs, or other plant-based substances, amino acids, or a mix of these constituents. Similarly, natural supplements refer to any supplement that is not artificially made in a laboratory and derives its nutritional value from products in nature. These products can be taken by mouth in different forms, are sold over the counter, and are advertised on the Internet with purported health claims. They are said to work by affecting the neurotransmitter systems that help people with symptoms of depression, anxiety, or insomnia. The Food Drug and Cosmetic Act treats dietary supplements like foods, not like drugs, so they must say on the label that they are dietary supplements.

#### 2.3.2. Anxiety, Depressive Symptoms, and Insomnia

Anxiety is characterized by a sensation of on-going worry that makes it difficult for a person to function. This can range from the temporary worry that a person has before an operation to the continuous anxiousness that is indicative of an anxiety disorder (e.g., generalized anxiety disorder, obsessive compulsive disorder, panic disorder, and social phobia) [14]. Debilitating physical symptoms of anxiety include, among others, migraines, uncontrollable trembling and perspiration, muscle tightness, and aches.

Depression is a frequent and dangerous mood illness, which is also known as major depressive disorder or clinical depression. People who are depressed have constant feelings of despair and hopelessness and they stop being interested in the things they used to like. In addition to the emotional challenges brought on by sadness, some people may also experience physical symptoms including persistent pain or digestive problems [15].

Insomnia is defined as having trouble falling asleep, staying asleep, waking up, or getting a good night’s sleep on a regular basis, even though there is enough time and opportunity to sleep, and this makes it hard to function during the day [16].

#### 2.3.3. Adults and Elderly

Adult individuals are defined as aged 18 and older. Elderly individuals are defined as aged 65 and older.

### 2.4. Data Extraction

Two authors (Y.A.A.-A. and D.K.) performed data extraction using a tabulated template form that was previously prepared by the authors. The table included relevant information for each study, including the following: (1) the first author and year of publication (e.g., reference); (2) location of the study (city and/or country); (3) study population of interest; (4) study sample size; (5) study design; (6) sleep health measurement tool; (7) sleep health domains; and (8) main findings summarized. Conflicts were discussed with a third author and resolved. Data were categorized and reported, in its tabulated form, according to health outcomes of concern (e.g., anxiety symptoms, depressive symptoms, and insomnia).

### 2.5. Data Presentation

Data were presented in its tabular form as described earlier and according to the outcome of interest. This review focused on three main health outcomes in relation to the intervention of concern. Mainly, the three health outcomes were used to initially report the data in three different tables (e.g., anxiety, depressive symptoms, and insomnia). Finally, and after inspecting the data and extracting it, a fourth table was created for studies that focused on more than a single health outcome of concern (e.g., effects on anxiety, depressive symptoms, and insomnia from the same dataset and reported in one single paper).

### 2.6. Quality Appraisal

Given that this review was restricted to studies that were RCTs by design, we opted to use the revised Risk of Bias tool (RoB2) to assess the quality of the included studies. RoB2 was developed by the Cochrane group and modified to ensure standardized scoring [17]. Using RoB2, the methodological quality of each study was independently assessed by two reviewers (Y.A.A.-A. and D.K.), with assessment scoring relating to selection bias, performance bias, attrition bias, and detection bias evidence, as well as the blinding of participants. Any conflicts between the two independent reviewers were resolved by a third senior investigator.

### 2.7. Ethical Approval

For this systematic review, and given the design of the paper, ethical approval was not applicable. This is because data utilized in this review were collected from previously published research in the literature. Studies included in this review that contributed to the dataset of this systematic review received ethical approval prior to data collection by their primary investigator.

## 3. Results

### 3.1. Search Results

As illustrated in Figure 1, our systematic search of the literature yielded 929 studies from PubMed, 1424 studies from Embase, 1420 studies from Medline, 577 studies from CINAHL, 93 studies from PsychINFO, and 159 studies from Cochrane. All the studies were exported to an Endnote library, after which all the duplicates (*n* = 1821) were detected and removed. A total of 2781 studies were independently screened by two authors (D.K. and Y.A.A.-A.) for the title and abstract for relevance to our research question and eligibility criteria. This step excluded a total of 2547 studies, either because they were not a randomized control trial or were not relevant to the research question of this systematic review. Following this step, full article screening was conducted by two authors for the remaining studies (*n* = 234). A total of 158 studies were excluded at this stage, mainly because the full text was unavailable (*n* = 18), they were not a randomized control trial (*n* = 22), they were not in the English language (*n* = 31), they were not peer reviewed (*n* = 14), or they were not original research reporting the results of the trial (*n* = 73). Finally, a total of 76 studies were included in this review.

### 3.2. Study Characteristics

Among the studies included in this review, the sample size ranged from 11 to 56,462 participants. The studies were conducted among a range of populations, including healthy adults, the elderly, and people with cognitive or non-cognitive chronic illnesses. The studies were conducted among a variety of countries across the globe, including USA, Australia, China, India, Iran, and the UK. Tables S2–S5 present a full list of all included studies and the relevant extracted data. Finally, Table S6 presents a synthetic summary of the main findings of each category of studies included in this systematic review (e.g., per health outcome), in addition to their implications for public health policy and clinical practice.

### 3.3. Quality Appraisal

The majority of studies that were included had a low risk of bias, as measured by the overall risk of bias score. According to the RoB 2 tool, the most consistent domains that decreased bias risk were performance bias (e.g., double blinding) and reporting bias (e.g., adequate selective outcome reporting), while selection bias (e.g., randomized selection in the population and adequate concealment of allocation to intervention) was the domain that increased bias risk in most of the studies. Table S7 presents the overall risk-of-bias assessment for the studies included in this review, reported for different domains according to the RoB2 risk assessment tool.

### 3.4. Study Presentation

Data obtained from included studies were tabulated and presented according to the health outcomes of interest. Four main categories were included in the presentation, including (1) studies reporting effects on anxiety symptoms only; (2) studies reporting effects on depressive symptoms only; (3) studies reporting effects on sleep health outcomes only; and (4) studies reporting effects on two or more of the relevant health outcomes.

### 3.5. Anxiety

A total of seven studies investigated anxiety symptoms as the primary outcome of interest. The sample size among those RCTs ranged from 20 participants in a USA-based study [18] to 180 participants in a Japanese-based study [19]. Overall, the results in the literature varied primarily by the type of plant-based and/or nutritional supplement received. Gonzalez et al., 2018, demonstrated that 28 days of a dietary supplement containing satiereal, naringin, and vitamin D3 did not have any detectable beneficial effects on anxiety [18]. However, among a sample of 180 healthy participants in Japan, hard capsules containing L-Lysine HCI and L-arginine significantly reduced anxiety symptoms, as measured by the cognitive stress battery and the state trait anxiety inventory [19]. Similarly, Australian, healthy young adults (*n* = 138) demonstrated significantly reduced stress, physical fatigue, and anxiety levels after receiving a multivitamin supplement containing high levels of vitamin B daily for 16 weeks [20]. Among a sample of 60 adults with increased weight (e.g., categorized as either overweight or obese) in Iran, synbiotics (e.g., 500 mg capsule containing Lactobacillus acidophilus, Lactobacillus casei, and Bifidobacterium bifidum plus inulin) were associated with a decrease in body weight, stress, and anxiety [21].

The majority of the included studies measured anxiety symptoms using questionnaires. Mainly, these tools included the Profile of Mood States (POMS) questionnaire and the State–Trait Anxiety Inventory questionnaire. However, some studies used objective methods to measure anxiety levels, including heart rate variability, actigraphy, [22] and salivary IgA [23].

### 3.6. Depressive Symptoms

A total of 26 studies investigated depressive symptoms as the primary outcome of interest. The sample size in those studies ranged from 11 participants in a USA-based study to 56,462 in a South Korean-based study. In the largest study conducted in South Korea, Nguyen et al. found that increasing the intake of multiple individual nutrients, fruits, and vegetables was associated with an improvement in depressive symptoms. Another study utilizing a large sample of 18,353 adults aged 50 years or older in the USA demonstrated that vitamin D3 supplementation did not affect the risk of depression or depressive symptoms, as measured by the eight-item Patient Health Questionnaire depression scale (PHQ-8) [24]. Similarly, vitamin C and folate supplementation were not associated with a change in depressive symptoms among a sample of 73 nursing home residents in the UK. Selenium levels, however, were significantly associated with depressive symptoms [25]. However, the results regarding the association between vitamin intake and depression remain mixed in the literature. For instance, Jorde et al. observed that receiving vitamin D supplementation for a year was associated with significantly lower scores on the Beck depression inventory (BDI) subscale among a sample of Norwegian adults [26]. This was also supported by the findings of Lee et al. among 3369 men aged 40–79 years across Europe. Furthermore, multivitamin supplements (e.g., vitamin B6, B12, and folic acid taken at a dosage of one pill every day for 12 weeks through the oral route) were shown to significantly decrease the depressive symptoms score measured using the Geriatric Depression Scale Short Form, the Korean version. Using the Montgomery–Asberg Depression Rating Scale (MADRS), depressive symptoms were shown to improve among a sample of 330 adult patients with major depressive disorders when receiving a combination capsule of reduced B vitamins [27]. This, in turn, provides evidence that B vitamins may be promising for treating and managing MDD.

### 3.7. Sleep Health Symptoms

A total of six studies investigated the effect of plant-based and nutritional supplements on sleep health. The number of participants in those studies ranged from 52 participants in a Japanese-based trial to 203 participants in a study conducted in Brazil. All the studies focused on sleep quality as a measure of sleep health outcomes, whereas no studies investigated other domains of sleep health (e.g., sleep duration, sleep problems, etc.). All studies measured sleep using self-reported measurement tools, including the Pittsburgh Sleep Index (PSQI), the Pittsburgh sleep diary tool, and the Richards Campbell Questionnaire Sleep (RCSQ). Overall, a number of supplements was found to be associated with better sleep quality, including natural melatonin, [28] saffron supplementation, [29] L-ornithine, [30] and vitamin D supplementation [31]. On the other hand, Ayurveda (a herbal preparation) was found to have no effect on sleep quality among 120 residents of elderly homes in India [32]. It is important to note that in some studies, sleep quality improved due to a supplement’s effect on an underlying medical condition that may have impacted sleep. For instance, among a sample of 105 male patients in China, Phellodendron Bawei tablets, when combined with α1-receptor blockers and 5α-reductase inhibitors, improved urinary tract symptoms more when compared to the placebo group receiving only α1-receptor blockers and 5α-reductase inhibitors treatment. In turn, this led to better sleep scores and an improvement of the outcomes [33].

### 3.8. Anxiety, Depressive, or Sleep Health Symptoms

A total of 41 studies investigated more than one health outcome as the primary outcome of concern in the trial (e.g., anxiety symptoms, depressive symptoms, and/or sleep health outcomes). The number of participants ranged from 29 participants in Japan [34] to 1000 participants in a study conducted in Netherlands, Spain, UK, and Germany [35]. In a study conducted in the Netherlands, no significant effect was found in relation to both anxiety and depressive symptoms when participants received vitamin D supplementation. Alternatively, Jamilian et al. reported that receiving vitamin D supplementation in addition to omega-3 fatty acids from fish oil resulted in a significant improvement in depressive symptoms, as measured by the Beck depression inventory scale, among 60 adults diagnosed with polycystic ovarian syndrome [36]. Similarly, combining a vitamin D supplement with probiotics demonstrated similar improvements on the Beck depression inventory scale among adults in Iran [37].

When receiving a multivitamin formulation containing numerous vitamin supplementation, Harris et al. reported an improvement in anxiety and depressive symptoms among 50 men in Australia. Hughes et al., 2020, reported similar findings among 39 young adults in the USA. When combining multivitamins with a mineral herbal supplement, participants reported an improvement in scoring on the depression anxiety stress scale (DASS) in Australia [38]. Among 60 adults diagnosed with a depressive disorder, it was shown that receiving a multivitamin combination (e.g., vitamins B1, B2, B3, B5, B6, and B12, and folate), combined with probiotics, was associated with significant improvement in anxiety and depressive symptoms [39]. Furthermore, other studies in the literature investigated probiotic supplementation on their own. For instance, Inoue et al., 2018, demonstrated that receiving probiotic supplementation for 12 weeks, when combined with resistance training, showed a significant decrease in anxiety and depressive symptoms scores, as compared to receiving only resistance training for 12 weeks. Receiving probiotics alone, without any combination, also showed improvement in anxiety symptoms among 30 university students in Malaysia. However, no significant effect was noted on depressive symptoms [40].

## 4. Discussion

This systematic review of the literature investigated the association between plant-based therapeutics and natural supplementations on anxiety symptoms, depressive symptoms, and sleep quality outcomes among adults and elderly populations from a diverse set of geographical and cultural contexts. With the cost of prescription drugs going up and the side effects that they can cause, more and more people are looking into herbal and other natural treatments for mental health problems. Mental disorders have continued to remain among the top ten leading causes of burdens worldwide, with no evidence of a global reduction since 1990 [41]. About 6.8 million people in the United States have generalized anxiety disorder. Furthermore, among those pursuing treatment, many must deal with the wide range of physical and mental side effects that often come with the treatment. Generally speaking, it may be plausible to claim that the public’s perception is that herbals or natural substances have fewer side effects, which may enable people to be more inclined to utilize such substances with less hesitantly when needed. Hence, there is a strong call to investigate natural anti-anxiety treatments that work well and may have a lower chance of side effects or withdrawal. Overall, our review provides insights on the current state of knowledge in the literature regarding the use of plant-based therapeutics and natural supplementations, and how such interventions can be utilized in clinical practice to improve health outcomes among both healthy and at-risk populations. This review provides qualitative evidence covering a wide range of populations, including those from different geographical contexts and different states of health.

Our study had several valuable findings that have the potential to inform clinical guidelines, policy-making, and intervention measures. Firstly, and in line with the previous literature on the topic, our review found positive evidence regarding the benefits of probiotic supplementation. A previous systematic review focused on clinical patients showed that probiotics may improve depression and anxiety symptoms [42]. Similarly, in another systematic review conducted by Smith et al., most studies demonstrated a significant relationship between probiotics and an improvement in depressive and anxiety symptoms [43]. In another review conducted by Wallace et al., most of the studies included found a positive association between receiving probiotics and depressive symptoms [44]. However, there exists very little literature currently regarding the most effective strains of probiotics, as well as the dosing and duration of treatments.

Moreover, we found that treatments containing amino acids, including L-lysine and L-arginine, were effective for improving anxiety and depressive symptoms. These findings are in line with other systematic reviews published on the topic. For example, Lakhan et al. demonstrated that L-lysine and L-arginine, when used in combination with herbal supplements containing extracts of passionflower or kava, were effective in improving anxiety-related symptoms, without the risk of serious side effects [45]. In regard to vitamin supplementation, we found that vitamin B complex supplementation improved anxiety symptoms, depressive symptoms, and sleep quality among a variety of populations. Among both healthy and at-risk cohorts, a systematic review conducted by Young et al. reported that although vitamin B supplementation benefited stress, there was no significant association with improvements in depressive or anxiety symptoms. Similarly, our review found no conclusive evidence regarding the association between vitamin D supplements. A systematic review and meta-analysis conducted by Cheng et al. confirmed the evidence that vitamin D supplementation can reduce negative emotions. This study provided insights that patients with major depressive disorder and individuals with vitamin D deficiency are most likely to benefit from supplementation [46]. However, it is important to interpret those results with caution due to the heterogeneity of the results. This supports the notion that further research is required to elucidate the relationship between vitamin D supplementation and the health outcomes of concern to our study. Regarding clinical guidelines, the World Federation of Societies of Biological Psychiatry (WFSBP) and the Canadian Network for Mood and Anxiety Treatments (CANMAT) Taskforce weakly recommend vitamin D supplementation for the treatment of psychiatric disorders.

This review provides a high level of evidence by synthesizing the published literature regarding the effectiveness of plant-based therapeutics and supplementation on anxiety symptoms, depressive symptoms, and sleep quality. This study is meant to provide insights regarding the findings of randomized control trials published on the topic and inform clinical guidelines about what supplements could be potentially beneficial in treatments. The findings of this review also have the potential to direct policy-making and intervention measures at the population level. To date, very few studies have reviewed the evidence in the literature regarding this topic. This review provides the most updated findings in the literature on the topic, including an abundance of research that was published in the past 5 years. Given the expected rise in negative mental and sleep health outcomes among the population following the pandemic and global lockdowns, there is a strong need to understand which supplements that are currently available to the public could aid in addressing mental and sleep health outcomes. The supplements and therapeutics identified in this study should be the target of intervention measures to increase their accessibility and affordability and allow them to be incorporated into clinical guidelines of treatment.

Despite the strengths of this study, it is important to interpret the results of this study with considerations to some noteworthy limitations. Firstly, most of the studies included in this review used survey-based tools to measure the health outcome of concern. Inherently, this approach is suitable due to the efficiency and feasibility of collecting data. However, it is important to note that self-reported surveys have several limitations, including social desirability bias and recall bias. Relating to measurement tools for health outcomes, it is important to note that individual studies are subject to confounding bias, where anxiety symptoms and sleep health symptoms could have a bidirectional relationship and influence each other, which in turn affects the results of the studies reviewed in this investigation. Furthermore, given the variability in perceptions of mental health in different cultural contexts, a volunteer bias must be acknowledged and considered. The selected studies in this review are subject to volunteer bias, where participants who actively decide to participate in the research may systematically differ from the general population. Moreover, our review may have been influenced by publication bias; unpublished studies on this subject may be more likely to have negative results, especially given the study designs of the included studies. Finally, our search strategy was limited to English language studies and did not include unpublished abstracts from conference proceedings or non-indexed journals. Future research should continue to investigate the therapeutic potential of plant-based and nutritional supplements, especially considering the projected increase in negative mental health outcomes following the COVID-19 pandemic. Finally, as we continue to improve our understanding of COVID-19 and its associated physical and mental symptoms, future research should investigate the association(s) between plant-based and natural supplements and COVID-19.

## 5. Conclusions

Overall, our systematic review of the literature highlighted the emerging evidence that demonstrates the benefits of probiotics and vitamin B complexes in association with anxiety symptoms, depressive symptoms, and sleep quality among healthy adults and the elderly. To the best of our knowledge, this is one of the first studies to have reviewed the evidence on the therapeutic impact of plant-based and nutritional supplements on anxiety, depressive, and sleep health symptoms. Future research should continue to investigate the therapeutic potential of plant-based and nutritional supplements, especially considering the projected increase in negative mental health outcomes following the COVID-19 pandemic.

## Data Availability

The authors declare that the data collected was gathered from publicly available databases and is available with this publication.

## Supplementary Materials

The following supporting information can be downloaded at: https://www.mdpi.com/article/10.3390/ijerph20065171/s1, Table S1. Keywords used in the systematic literature search; Table S2. Studies investigating the effect of plant-based therapeutics and natural supplements on anxiety symptoms; Table S3. Studies investigating the effect of plant-based therapeutics and natural supplements on depressive symptoms; Table S4. Studies investigating the effect of plant-based therapeutics and natural supplements on sleep health outcomes; Table S5. Studies investigating the effect of plant-based therapeutics and natural supplements on multiple outcomes (at least two of the three main outcomes of interest e.g., anxiety symptoms, depressive symptoms, and/or sleep health outcomes); Table S6. Synthetic summary of the main findings of each category of studies included in this systematic review (e.g., per health outcome), in addition to their implications for public health policy and clinical practice. Table S7. Overall risk-of-bias assessment for studies included in this review, presented for different domains according to the RoB2 risk assessment tool. References [18,19,20,21,22,23,24,25,27,28,29,30,31,32,33,34,35,36,37,38,39,40,47,48,49,50,51,52,53,54,55,56,57,58,59,60,61,62,63,64,65,66,67,68,69,70,71,72,73,74,75,76,77,78,79,80,81,82,83,84,85,86,87,88,89,90,91,92,93,94,95,96,97,98,99,100] are cited in the supplementary materials.
