# Supplementary material for: The Therapeutic Impact of Plant-Based and Nutritional Supplements on Anxiety, Depressive Symptoms and Sleep Quality among Adults and Elderly: A Systematic Review of the Literature
Source: Int J Environ Res Public Health. 2023 Mar 15;20(6):5171. doi: 10.3390/ijerph20065171 (PMC10049309; doi:10.3390/ijerph20065171)
Supplement: Supplementary file 1 [file ijerph-20-05171-s001.zip › ijerph-2249468-supplementary.pdf]

Table S1. Keywords used in the systematic literature search

1) Intervention

('diet supplementation' OR 'nutrition supplement' OR 'Chinese medicine' OR 'herbal supplements' OR 'alternative therapies' OR supplementation OR 'non prescription drug' OR Ayurveda OR vitamin OR mineral OR herb)

AND

2) Outcome of Interest - Anxiety, depression and insomnia

(anxiety OR 'anxiety disorder' OR depression OR 'major depression' OR insomnia)

AND

3) Population of Interest - Adults and Elderly

(adult OR aged OR elderly OR senior)

Table S2. Studies investigating the effect of plant-based therapeutics and natural supplements on anxiety symptoms.

Reference	Location of Study	Population	Sample Size	Intervention	Outcome reported	Tool for Outcome Measurement	Summary of Main Findings
Giles et al., 2015 [1]	USA	72 young adult females	N= 72	2800 mg/day fish oil [Omega-3 fatty acid] (n = 36, 23 females) or olive oil control (n = 36, 22 females) for 35 days.	Anxiety symptoms	(i) Profile of Mood States (POMS) Questionnaire (ii) State–Trait Inventory for Cognitive and Somatic Anxiety (iii) Trier Social Stress Test (TSST) (iv) Emotional Interference Task (EIT) (v) Morphed Faces Task (MFT) (vi) Salivary cortisol and interleukin-1β	Rated anger and confusion increased with stress in the olive oil group but remained stable in the fish oil group. However, fish oil had no further effects on mood, cognitive function, cortisol, or IL-1β. Fish oil exerted few effects in stressful and non-stressful situations
Gonzalez et al., 2018 [2]	USA	Twenty healthy adults (25.5 ± 3.8 years; 87.3 ± 20.7 kg; 169.9 ± 10.6 cm; 29.9 ± 5.1 body mass index)	N=20	Ten participants were provided with a dietary supplement containing 178 mg satiereal, 100 mg naringin, and 2,000 IU vitamin D3 daily (SUPP),	Anxiety symptoms	Profile of mood states, visual analog scales, modified trait food-cravings questionnaire, and a modified state food-cravings questionnaire.	No significant differences between groups were observed for mood states, subjective measures of food cravings, or feelings of anxiety, fullness, bloating, hunger, craving, and stress (p >.05).

				and ten participants were provided a placebo (PL) for 28 days			
Hadi et al., 2019 [3]	Iran	60 adults with overweight or obesity	N=60	Participants were randomly assigned into two groups to receive either synbiotics (n = 30) in form of a 500 mg capsule (containing Lactobacillus acidophilus, Lactobacillus casei and Bifidobacterium bifidum plus inulin) or placebo (n = 30) for 8 weeks.	Anxiety symptoms	The level of total cholesterol (TC), triglycerides (TG), low-density lipoprotein cholesterol (LDL-C), high-density lipoprotein cholesterol (HDL-C), fasting blood glucose (FPG), insulin, body weight, body mass index (BMI), waist circumference (WC), systolic blood pressure (SBP), diastolic blood pressure (DBP), stress, anxiety, and depression were measured at the baseline and end of the study.	A significant between-group decrease in body weight ($P = 0.03$), TC ($P = 0.01$), TG ($P = 0.02$), LDL-C ($P = 0.01$), stress ($P < 0.001$), anxiety ($P = 0.03$), and depression ($P = 0.03$) was found in the synbiotic group compared to the placebo. However, synbiotics had no significant effect on HDL-C, SBP, DBP, FPG and fasting insulin concentrations, as well the BMI and WC ($P < 0.05$).
Jaatinen et al., 2014 [4]	Finland	67 participants aged 18–63 years	N=67	Yogurt enriched with alpha-lactalbumin, casein tripeptides and	Anxiety symptoms	State-Trait Anxiety Inventory, Profile of Mood States, salivary cortisol, inflammatory	Higher ratings of vigor ($p = 0.047$) and reduced feeling of inefficiency ($p = 0.048$) in the active group were observed. HRV (baseline adjusted mean 49.1 ± 2.3 ms) and recovery index (106.6 ± 33.4) were higher in the active group than in controls

				B vitamins (active) or isoenergetic standard yogurt (control)		markers, blood pressure, heart rate variability (HRV) and actigraphy	(42.5 ± 2.2 ms and 80.0 ± 29.3) (p = 0.046 and p = 0.02, respectively).
Noorwali et al., 2017 [5]	UK	sixty participants aged 18–40 years	N=60	Either probiotic (Lactobacillus acidophilus CUL60 and CUL21, Bifidobacterium lactis CUL34, Bifidobacterium bifidum CUL20) or placebo (maltodextrin) capsules.	Anxiety symptoms	(i) State-Trait Anxiety Inventory questionnaire (ii) Remember, Know, Guess task (iii) visual analogue scale (iv) salivary IgA	When comparing treatment groups in terms of EM, the probiotic group recalled significantly more negative images compared to placebo (P = 0·022). This effect was more pronounced in female participants (P = 0·009). For male participants only, pain threshold was significantly reduced at the second assessment under the placebo condition (−3·687 seconds, P = 0·027) but not following probiotic administration, potentially indicating a protective effect of probiotic treatment on pain threshold. No further significant results were observed.
Pipingas et al., 2013 [6]	Australia	138 healthy young adult participants between the ages of 20 and 50 years over a 16-week period	N=138	A Multivitamin supplement containing high levels of B-vitamins was administered daily	Anxiety Symptoms	Chronic mood measures (GHQ-28, POMS, STAI-S, Chalder fatigue, PILL, Bond-Lader and custom visual analogue scales) were administered pre-dose at baseline, 8- and 16-weeks.	At-home mobile-phone assessments, where assessments were conducted post-dose, revealed significantly reduced stress, physical fatigue and anxiety in the MV group in comparison to placebo across a number of time points.
Smriga et al., 2007 [7]	Japan	180 healthy participants (male=54, female=54) (22 to 59 years)	N= 180	Test group (Lys/Arg group) received hard capsules (Aliment Co.,	Anxiety symptoms	(i)Cognitive Stress Battery (ii) State Trait Anxiety Inventory (STAI)	Without regard to gender, the amino acid treatment significantly reduced both state anxiety and trait anxiety induced by cognitive stress battery.

				Ltd, Tokyo, Japan) containing L-Lysine HCI and L-arginine (Ajinomoto Co., Inc., Tokyo, Japan) and were instructed to ingest the capsules twice daily (at breakfast and dinner) during 7 consecutive days. The subjects assigned to placebo group received hard capsules containing tapioca starch (3.00 g * 2/day)			

Table S3. Studies investigating the effect of plant-based therapeutics and natural supplements on depressive symptoms

Reference	Location of Study	Population	Sample Size	Intervention	Outcome reported	Tool for Outcome Measurement	Summary of Main Findings
Ghorbani et al., 2018 [8]	Iran	40 adult outpatients, who met the diagnostic criteria of moderate depression according to DSM-V	N=40	Patients received fluoxetine (20 mg/d) for 4 weeks. Then, either a synbiotic capsule (plus fluoxetine) or placebo (plus fluoxetine) was added to the therapy for 6 weeks	Depressive symptoms	Hamilton rating scale for depression (HAM-D)	There was a greater reduction in HAM-D score in synbiotic treated patients (Mean ±SD = -19.25 ±1.71) compared to placebo taking group (Mean ±SD = -17.75 ±2.05; P = 0.024). At the end of the 10th week of the intervention, it was found that the synbiotic group had a significantly decreased HAM-D score compared to the placebo (3.65 vs. 4.80, P = 0.013).
Gosney et al., 2008 [9]	UK	Seventy-three residents from eleven nursing and residential homes on Merseyside	N=73	Selenium, vitamin C and folate provided for 8 weeks	Depressive symptoms	e Hospital Anxiety and Depression rating scale (HAD), and MADRS	Depression was significantly associated with selenium levels, but not with folate or vitamin C levels. No individual with a HAD depression score of >or=8, had selenium levels >1.2 microM. In those patients with higher HAD depression scores, there was a significant reduction in the score and a significant increase in serum selenium levels after 8 weeks of micronutrient supplementation. Placebo group scores were unchanged. This small study concluded that depression was associated with low levels of selenium in frail older individuals. Following 8 weeks of micronutrient supplementation, there was a significant increase in selenium levels and

							improved symptoms of depression occurred in a subgroup.
Imaoka et al., 2019 [10]	Japan	67 participants aged 60 years or more with non-cognitive dysfunction	N= 67	An exercise group (Ex group, n = 36) and an exercise plus nutrition group (Ex+Nt group, n = 31). The Ex group completed a memory training activity for 15 mins and aerobic exercise for 45 mins once a week for 90 days. The Ex+Nt group completed the same training plus received soy peptide for 90 days.	Depressive symptoms	The Mini-Mental Status Examination score, trail-making test A/B score, skeletal muscle mass index, grip strength, gait speed, and geriatric depression scale -15 were measured at baseline and post intervention.	A two-way analysis of variance revealed significant time effects on trail-making test-A score, skeletal muscle index, grip strength, geriatric depression and gait speed in both groups.
Jorde et al., 2008 [11]	Norway	441 subjects (body mass index 28–47 kg m^{-2}, 159 men and 282 women, aged 21–70 years)	N=441	20.000 or 40.000 IU vitamin D per week versus placebo for 1 year.	Depressive symptoms	Beck Depression Inventory (BDI) score with subscales 1–13 and 14–21.	Subjects with serum 25(OH)D levels <40 nmol L^{-1} scored significantly higher (more depressive traits) than those with serum 25(OH)D levels ≥40 nmol L^{-1} on the BDI total [6.0 (0–23) versus 4.5 (0–28) (median and range)] and the BDI subscale 1–13 [2.0 (0–15) versus 1.0 (0–29.5)] ($P < 0.05$). In the two groups given vitamin D, but not in the placebo group, there was a significant improvement in BDI scores after 1 year.
Kaviani et al.,	Iran	56 subjects with	N= 56	Intervention	Depressive	Biochemical	Following intervention, significant changes

2020 [12]		mild to moderate depression, aged 43.0 ± 1.15years		(50,000 IU cholecalciferol/2wks) and control (placebo).	symptoms	parameters (serum 25(OH)D, iPTH, oxytocin and platelet serotonin), and depression severity (Beck Depression Inventory-II (BDI-II[1])) were initially and finally assessed.	were observed in the intervention group compared to the controls: 25(OH)D concentrations increased (+40.83±28.57 vs. +5.14±23.44 nmol/L, $P<0.001$) and BDI scores decreased (-11.75±6.40 vs. -3.61±10.40, $P = 0.003$).
Kazemi et al., 2019 [13]	Iran	One hundred and ten depressed patients	N= 110	The probiotic (*Lactobacillus helveticus* and *Bifidobacterium longum*), prebiotic (galactooligosaccharide) or placebo for 8 weeks.	Depressive symptoms	Beck Depression Inventory (BDI) score as a primary outcome as well as the kynurenine/tryptophan ratio and tryptophan/branch chain amino acids (BCAAs) ratio as secondary outcomes	From baseline to 8 weeks, probiotic supplementation resulted in a significant decrease in BDI score (18.25–9.0) compared to the placebo (18.74–15.55) and prebiotic (19.43–14.14) supplementation (p = 0.042).
Krikorian et al., 2013 [14]	USA	26 older adults	N= 26	Either chromium picolinate (CrPic) or placebo for 12 weeks.	Depressive symptoms	Memory and depression were assessed prior to treatment	Although learning rate and retention were not enhanced by CrPic supplementation, we observed reduced semantic interference on learning, recall, and recognition memory

						initiation and during the final week of treatment.	tasks. These findings suggest that supplementation with CrPic can enhance cognitive inhibitory control and cerebral function in older adults at risk for neurodegeneration.
Lee et al., 2008 [15]	Europe	3,369 men aged 40–79 years	N= 3369	25-hydroxyvitamin D (25(OH)D) levels	Depressive symptoms	Cognitive function was assessed using the Rey–Osterrieth Complex Figure (ROCF) test, the Camden Topographical Recognition Memory (CTRM) test and the Digit Symbol Substitution Test (DSST). Serum 25(OH)D levels were measured by radioimmunoassay. Additional assessments included measurement of physical activity, functional performance	After adjusting for additional confounders, 25(OH)D levels were associated with only score on the DSST (β per 10 nmol/l=0.152; 95% CI 0.051 to 0.253).

						and mood/depression.	
Lee et al., 2016 [16]	South Korea	Forty-eight adults 65 years of age and older with Mild Cognitive Impairment (experimental, *n* = 24; control, *n* = 24)	N= 48	Multivitamin supplements as experimental treatment consisted of vitamin B6, B12, and folic acid taken at a dosage of one pill every day for 12 weeks through the oral route.	Depressive symptoms	Measures were Mini Mental State Examination-Korean, serum homocysteine level, and Geriatric Depression Scale Short Form Korea Version.	There were significant effects of multivitamin supplements on cognitive function (F = 3.624, *p* = .021), serum homocysteine level (F = 6.974, *p* = .001), and depression (F = 10.849, *p* = .001).
Lespérance et al., 2011 [17]	Canada	432 Adult outpatients with Major Depressive Episode	N= 432	8 weeks of 1,050 mg/d of eicosapentaenoic acid (EPA) and 150 mg/d of docosahexaenoic acid (DHA) or matched sunflower oil placebo (2% fish oil).	Depressive symptoms	The primary outcome was the self-report Inventory of Depressive Symptomatology (IDS-SR30); the secondary outcome was the clinician-rated MADRS.	The adjusted mean difference between treatment and placebo was 1.32 points (95% CI, -0.20 to 2.84; P = .088) on the IDS-SR30 and 0.97 points (95% CI, -0.012 to 1.95; P = .053) on the MADRS. Planned subgroup analyses revealed a significant interaction of comorbid anxiety disorders and study group (P = .035). For patients without comorbid anxiety disorders (n = 204), omega-3 supplementation was superior to placebo, with an adjusted mean difference of 3.17 points on the IDS-SR30 (95% CI, 0.89 to 5.45; P = .007) and 1.93 points (95% CI, 0.50 to 3.36; P = .008) on the MADRS.
Lucas et al., 2008 [18]	Canada	120 Women with moderate-to-severe PD	N=120	1.05 g E-EPA/d plus 0.15 g ethyl-docosahexaenoic acid/d (n = 59) or placebo (n = 61)	Depressive Symptoms	(i) Psychological General Well-Being Schedule (PGWB)], (ii)[20-item	Stratification analyses for MDE diagnosis at baseline indicated that differences in adjusted 8-wk changes between the E-EPA group without MDE (n = 46) and the placebo group (n = 45) were 8.0 (95% CI: 0.6, 15.3; P = 0.034)

				for 8 wk.		Hopkins Symptom Checklist Depression Scale (HSCL-D-20), (iii) 21-item Hamilton Depression Rating Scale (HAM-D-21)]	for the PGWB, −0.2 (95% CI: −0.01, −0.4; P = 0.040) for the HSCL-D-20, and −2.7 (95% CI: −0.3, −5.1; P = 0.030) for the HAM-D-21. Differences in adjusted 8-wk changes between the E-EPA group with MDE (n = 13) and the placebo group (n = 16) were not significant.
Mech & Farah, 2016 [19]	USA	330 adult patients with MDD (*DSM-5*) and positive for either *MTHFR* C67 7T or A1298C polymorphism	N= 330	160 patients received placebo, while 170 received a capsule containing a combination of reduced B vitamins.	Depressive symptoms	The MADRS was used to evaluate efficacy for MDD.	A combination of reduced B vitamins and micronutrients, when used in the treatment of MDD in patients with *MTHFR* polymorphism, resulted in a separation from placebo by week 2, and 42% of the treatment arm achieved remission by week 8.
Moore et al., 2018 [20]	Ireland	249 adults aged 70 years and older	N= 249	A supplement containing folic acid (400 μg), vitamin B12 (10 μg), vitamin B6 (10 mg) and riboflavin (10 mg) or placebo daily for 2 years.	Depressive symptoms	Cognitive function was assessed before and after the 2-year intervention using the Frontal Assessment Battery (FAB) and the Repeatable Battery of the Assessment of Neuropsycholo	In the case of depression, although the effect of B-vitamin intervention on depression (i.e. CES-D score) did not reach statistical significance, rates of depression (i.e. % with CES-D score > 16) doubled over the 2-year period in the placebo group (from 4·0% to 8·0%) compared with a minimal increase in the B-vitamin group (from 4·0% to 4·8%)

						gical Status (RBANS). Depression was assessed using the Centre for Epidemiological Studies Depression (CES-D) scale.	
Ng et al., 2017 [21]	China	246 Participants aged 65 and above	N= 246	24 weeks duration interventions with nutritional supplementation (N=49), physical training (N=48), cognitive training (N=50), combination intervention (N=49) and usual care control (N=50).	Depressive symptoms	Geriatric Depression Scale (GDS-15)	Estimated 12-month cumulative incidence of depressive symptoms (GDS≥2) relative to control were OR=0.38, p=0.037 (nutrition); OR=0.71, p=0.40 (cognitive); OR=0.39, p=0.042 (physical training) and OR=0.38, p=0.037 (combination). Changes in gait speed and energy level were significantly associated with changes in GDS scores over time.
Nguyen et al., 2021 [22]	South korea	56,462 adults aged 18 years	N= 56,462	Dietary factors, including intakes of multiple individual nutrients, fruits, and vegetables, were assessed.	Depressive symptoms	N/A	In both women and men, high fruit or vegetable consumption was linked with a lower risk of T2DM, hypertension, dyslipidemia, osteoarthritis, and depression than low consumption.
Okereke et al., 2020 [23]	USA	18,353 adults aged 50 years or older without depression or clinically	N=18,353	Vitamin D_3 (2000 IU/d of cholecalciferol) and fish oil or	Depressive symptoms	8-item Patient Health Questionnaire depression scale	Risk of depression or clinically relevant depressive symptoms was not significantly different between the vitamin D_3 group (609 depression or clinically relevant depressive

		relevant depressive symptoms		placebo; 9181 were randomized to vitamin D_3 and 9172 were randomized to matching placebo.		[PHQ-8]	symptom events; 12.9/1000 person-years) and the placebo group (625 depression or clinically relevant depressive symptom events; 13.3/1000 person-years) (hazard ratio, 0.97 [95% CI, 0.87 to 1.09]; $P = .62$); there were no significant differences between groups in depression incidence or recurrence. No significant differences were observed between treatment groups for change in mood scores over time; mean change in PHQ-8 score was not significantly different from zero (mean difference for change in mood scores, 0.01 points [95% CI, −0.04 to 0.05 points]).
Parletta et al., 2017 [24]	Australia	152 eligible adults aged 18–65 were recruited ($n = 95$ completed 3-month and $n = 85$ completed 6-month assessments)	N=152	Fortnightly food hampers and MedDiet cooking workshops for 3 months and fish oil supplements for 6 months, or attend social groups fortnightly for 3 months.	Depressive symptoms	Assessments at baseline, 3 and 6 months included mental health, quality of life (QoL) and dietary questionnaires, and blood samples for erythrocyte fatty acid analysis.	At 3 months, the MedDiet group had a higher MedDiet score ($t = 3.95$, $P < 0.01$), consumed more vegetables ($t = 3.95$, $P < 0.01$), fruit ($t = 2.10$, $P = 0.04$), nuts ($t = 2.29$, $P = 0.02$), legumes ($t = 2.41$, $P = 0.02$) wholegrains ($t = 2.63$, $P = 0.01$), and vegetable diversity ($t = 3.27$, $P < 0.01$); less unhealthy snacks ($t = -2.10$, $P = 0.04$) and red meat/chicken ($t = -2.13$, $P = 0.04$). The MedDiet group had greater reduction in depression ($t = -2.24$, $P = 0.03$) and improved mental health QoL scores ($t = 2.10$, $P = 0.04$) at 3 months. Improved diet and mental health were sustained at 6 months. Reduced depression was correlated with an increased MedDiet score ($r = -0.298$, $P = 0.01$), nuts ($r = -0.264$, $P = 0.01$), and vegetable diversity ($r = -0.303$, $P = 0.01$).
Rice et al., 2014 [25]	Australia	400 participants (15- to 25-year-old)	N=400	12 weeks of treatment with	Depressive symptoms	Montgomery-Asberg	Because of using a large sample, results from this study will provide the strongest evidence

		presenting with major depressive disorder.		omega-3 PUFAs plus cognitive behavioural case management compared with treatment with placebo plus cognitive behavioural case management.		Depression Rating Scale	to date to inform the use of omega-3 PUFAs as first-line therapy in young people presenting with major depressive disorder.
Rizzo et al., 2012 [26]	Italy	46 depressed females aged 66-95years, diagnosed with depression according to DSM-IV	N=46	22 depressed females were included in the intervention group (2.5 g/day of n-3 PUFA for 8 weeks), and 24 in the placebo group	Depressive symptoms	Geriatric Depression Scale (GDS), blood fatty acid composition and erythrocyte phospholipids	The mean GDS score and AA/EPA ratio, in whole blood and RBC membrane phospholipids, were significantly lower after 2 months supplementation with n-3 PUFA.
Rondanelli et al., 2011 [27]	Italy	Forty-six depressed females, aged 66–95 years.	N=46	22 depressed females were included in the intervention group (n-3 group, that received 2.5 g/day of n-3 LCPUFA, with 1.67 grams of EPA and 0.83 grams of DHA), and 24 patients in the placebo group.	Depressive symptoms	Geriatric Depression Scale (GDS) and Short-Form 36-Item Health Survey (SF-36)	The mean GDS at 2 months was significantly lowered only for the n-3 group. SF-36 physical and mental components were significantly increased in the intervention group.
Rondanelli et al., 2011 [28]	Italy	Forty-one patients (58.5% women;	N=41	oral essential amino acids 4 gr 2	Depressive symptoms	Depressive symptoms	Compared with the placebo group, EAA patients improved nutrition (MNA score

		mean age 79.8 yrs) with sequelae of coronary artery disease (73%), femoral fracture (34%)		times a day for 8 weeks or isocaloric placebo.		(Geriatric Depression Scale, GDS), nutritional panel (Mini Nutritional Assessment, MNA; serum albumin and prealbumin levels), muscle strength (Hand Grip, HG), Activity Daily Life (ADL), Quality of Life (SF-36, HRQoL) and amino acid profile.	22.6 ± 1.5 post *vs* 21.8 ± 1.6 pre; $p < 0.04$, albumin g/dl 4.04 ± 0.35 post *vs* 3.88 ± 0.3 pre; $p < 0.01$), GDS(score 10.3 ± 1.75 post vs 13.85 ± 3.37 pre; $p < 0.001$), HG (Kg 19.75 ± 1.7 post *vs* 18.68 ± 1.36 pre; $p = 0.001$), ADL ($p < 0.04$) and both physical and mental components of SF-36 ($p < 0.002$).
Moghaddam et al.,2017 [29]	Iran	96 adults who aged 60 years	N= 96	Group A received fish four times a week whereas their peers in group B consumed fish two or fewer times per week.	Depressive symptoms	12-item general health questionnaire (GHQ-12) and 15-item Geriatric Depression scale (GDS-15).	The results indicated that GHQ and GDS scores decreased non-considerably following three-month intervention. Fish consumption was associated with a lower likelihood of depression (dichotomized GDS: OR[odds ratio] = 0.73, 95% CI[confidence interval] = 0.28-1.87, p = 0.618; dichotomized GHQ: OR = 0.67, 95% CI = 0.26-1.73; p = 0.334).
Shinto et al., 2016 [30]	America	31 participants	N= 31	Omega-3 fatty acids at six grams per day over three months.	Depressive symptoms	MADRS	Improvement on MADRS between groups was not significantly different at the 3-month end point with 47.4% in the omega-3 fatty acid group and 45.5% in the placebo group showing

							50% or greater improvement ($p = 0.30$). Omega-3 fatty acids as an augmentation therapy for treatment-resistant depression in MS was not significantly different than placebo in this pilot trial. Omega-3 fatty acid supplementation at the dose given was well-tolerated over 3 months.

Table S4. Studies investigating the effect of plant-based therapeutics and natural supplements on sleep health outcomes.

Reference	Location of Study	Population	Sample Size	Intervention	Outcome reported	Tool for Outcome Measurement	Summary of Main Findings
Gandolfi et al., 2020 [31]	Brazil	203 adult patients who were admitted to the ICU and administered with analgesics and/or sedatives.	N= 203	Oral melatonin (10 mg) or placebo for up to seven consecutive nights.	Sleep Quality	(i)The number of observed sleeping hours at night was assessed by the bedside nurse. (ii)Sleep quality was evaluated using the Richards Campbell Questionnaire Sleep (RCSQ). (iii) Melatonin levels were determined by enzyme-linked immunosorbent assay.	Sleep quality was assessed to be better in the melatonin group than that in the placebo group with a mean (SD) of 69.7 (21.2) and 60.7 (26.3), respectively ($p = 0.029$).
Gong et al., 2021 [32]	China	105 male patients	N=105	The standard management (SM) group was treated with tamsulosin + finasteride + placebo, while the	Sleep Quality	Age, body mass index (BMI), blood prostate-specific antigen (PSA), PV, Qmax, IPSS; IPSS voiding subscore (IPSS-V), IPSS storage	Phellodendron Bawei tablets combined with α1-receptor blockers and 5α-reductase inhibitors can improve lower urinary tract symptoms associated with urine storage, sleep quality, and medication compliance in patients with benign prostatic hyperplasia compared to placebo with standard management.

				experimental group was treated with tamsulosin + finasteride + Phellodendron Bawei tablets.		subscore (IPSS-S), and IPSS quality of life (IPSS-QOL)], five-item version of the International Index of Erectile Function (IEFF-5) score, Athens Insomnia Scale (AIS) score, and the Medication Adherence Questionnaire (MAQ)	
Lopresti et al., 2021 [33]	Australia	120 adults	N=120	Either a placebo, 14 mg, or 28 mg of a standardised saffron extract (affron®), 1 h before bed for 28 days	Sleep Quality	Outcome measures included the Pittsburgh Sleep Diary (with sleep quality ratings as the primary outcome measure), Insomnia Symptom Questionnaire (ISQ), Profile of Mood States, Restorative	Compared to the placebo, saffron supplementation was associated with greater improvements in sleep quality ratings (primary outcome measure), mood ratings after awakening, the ISQ total score, and ISQ-insomnia classifications. However, there were no significant differences between the saffron and placebo groups in other questionnaire and sleep diary outcome measures. Sleep improvements were similar for the two administered saffron doses.

						Sleep Questionnaire, the Functional Outcomes of Sleep Questionnaire, and evening salivary melato nin and cortisol concentrations.	
Manjunath & Telles, 2005 [34]	India	120 residents from a home for the aged	N= 120	Yoga (physical postures, relaxation techniques, voluntarily regulated breathing and lectures on yoga philosophy), Ayurveda (a herbal preparation), and Wait-list control (no intervention).	Sleep Quality	self-assessment of sleep	The Yoga group showed a significant decrease in the time taken to fall asleep (approximate group average decrease: 10 min, P<0.05), an increase in the total number of hours slept (approximate group average increase: 60 min, P< 0.05) and in the feeling of being rested in the morning based on a rating scale (P<0.05) after six months. The other groups showed no significant change.
Miyake et al., 2014 [35]	Japan	Fifty-two apparently healthy Japanese adults	N= 52	The L-ornithine (400 mg/day) or placebo group for 8 weeks.	Sleep Quality	Perceived mood and quality of sleep were measured by the Profile of Mood States	Serum cortisol levels and the cortisol/DHEA-S ratio were significantly decreased in the L-ornithine group in comparison with the placebo group. Also, anger was reduced and perceived sleep quality was improved in the L-ornithine group.

						(POMS), Athens Insomnia Scale (AIS), and Ogri-Shirakawa-Azumi sleep inventory MA version (OSA-MA).	
Majid et al., 2017 [36]	Iran	89 people with sleep disorders based on Petersburg's Sleep Index.	N= 89	Intervention group received a 50 000-unit vitamin D supplement, one in a fortnight for 8 weeks. Meanwhile, placebo group received placebo.	Sleep Quality	Petersburg's Sleep Quality Questionnaire, International Physical Activity Questionnaire, general information questionnaire, sun exposure, vitamin D serum level and 3-day food record questionnaire	Sleep score (PSQI) reduced significantly in vitamin recipients as compared with placebo recipients ($P < 0.05$). This difference was significant even after modifying confounding variables ($P < 0.05$).

Table S5. Studies investigating the effect of plant-based therapeutics and natural supplements on multiple outcomes (at least two of the three main outcomes of interest e.g., anxiety symptoms, depressive symptoms, and/or sleep health outcomes)

Reference	Location of Study	Population	Sample Size	Intervention	Outcome reported	Tool for Outcome Measurement	Summary of Main Findings
Kim et al., 2021 [37]	South Korea	Thirty adults with Major Depressive Disorder	N= 30	The Treatment Group was treated with electroacupuncture plus moxibustion, and the Control Group received sham interventions at non-acupoints for 8 weeks.	Depressive symptoms, anxiety symptoms and insomnia	The primary outcome measure was the intergroup difference of the mean change of total score of the Hamilton rating scale for depression (HRSD) between baseline and week 9. Secondary outcome measures were Beck's depression inventory, insomnia severity index, the state-trait anxiety inventory, the EuroQol-5 dimension index, the measure yourself medical outcome profile version 2, and frontal alpha asymmetry measured by electroencephalography . Adverse events (AEs) were monitored for safety assessment.	The primary outcome measure was not significantly different between the two groups (p=0.2641), although the scores of HRSD in both groups improved significantly after treatment. No significant difference was identified between groups in secondary outcome measures. The incidence of AE was not significantly different between the two groups (p=0.1067).

Koning et al., 2019 [38]	Netherlands	155 participants aged 60–80 y who had clinically relevant depressive symptoms	N= 155	Participants received 1200 IU/d vitamin D_3 (n = 77) or placebo tablets (n = 78) for 12 months	Depressive symptoms and Anxiety symptoms	(i)Depressive symptoms were assessed using the CES-D scale. (ii)Functional limitations were assessed using the Longitudinal Aging Study Amsterdam (LASA) Functional Limitations questionnaire. (iii)Physical performance was assessed using a modified version of the Short Physical Performance Battery (SPPB). (iv)The presence of Major Depressive Disorder was assessed using the depression section of the Composite International Diagnostic Interview. (v)Anxiety was assessed using the Beck Anxiety Inventory (BAI) (25). (vi)Cognitive function was assessed using the Stroop Color-Word Test (26). (vii)HR-QoL was assessed using the EuroQol-5 Dimensions	The supplementation increased serum 25(OH)D concentrations in the intervention group to a mean ± SD of 85 ± 16 nmol/L compared with 43 ± 18 nmol/L in the placebo group after 6 mo ($P < 0.001$). No relevant differences between the treatment groups were observed regarding depressive symptoms, functional limitations, physical performance, anxiety symptoms, cognitive functioning, mobility, handgrip strength, and health-related quality of life.

						(EQ-5D) and the Short Form–36 Health Survey (SF-36). (viii)The timed up-and-go (TUG) test was assessed to test functional mobility. (ix)Muscle strength was measured with a handgrip strength test using a strain-gauged dynamometer.	
Harris et al., 2011 [39]	Australia	fifty men, aged 50–69 years	N= 50	Multivitamin formulation that contained vitamins (at levels above recommended daily intakes), minerals, antioxidants, and herbal extracts, or a placebo for 8 weeks	Anxiety and depressive symptoms	DASS-21 (Depression, Anxiety and Stress Scale)	Compared with placebo, there was a significant reduction in the overall score on a DASS-21 and an improvement in alertness and general daily functioning in the multivitamin group.
Hegde et al., 2020 [40]	India	Thirty adults (males = 14, females = 16) admitted in a residential holistic health care center with an age range of 29.66 ± 6.63 years and diagnosis of one of the Anxiety	N= 30	Integrated yoga-based lifestyle program (YT; *n* = 15) and (2) YT + Ayurveda (YA; *n* = 15).	Anxiety symptoms and Sleep Quality	Clinical symptoms (HAM-A, State and Trait Anxiety Inventory, and Brief Psychiatric Rating Scale), sleep quality (sleep rating questionnaire), and cognition (Stroop test and digit letter substitution test)	Within-group comparisons showed a significant improvement in clinical symptoms, cognition and sleep quality in both the groups. Between-group comparisons showed significantly better scores in Stroop word task for YA group as compared to YT group. Furthermore, there was a trend toward better improvement in sleep

		Disorders (generalized AD, *n* = 18; social phobia, *n* = 8; and panic AD, *n* = 4)					quality for YA group.
Hoffman et al., 2020 [41]	Israel	Sixty recreationally trained men (n = 48) and women (n = 12), between 20 and 35 years	N= 60	Sceletium tortuosum supplementation and placebo	Anxiety and Depressive symptoms	Visual analog scale (VAS), Profile of Mood States questionnaire and reactive agility and visual tracking assessments	Significant improvements were noted for Sceletium Tortuosum in complex reactive performance that required subjects to respond to repeated visual stimuli with a cognitive load compared with Placebo. However, no significant changes were noted between the groups in either VAS or total mood score. In addition, no differences were observed in simple reaction assessments. The results of this study demonstrate an ergogenic benefit in complex reactive tasks that include a cognitive load. However, in this subject population studied, no benefits in mood were observed.
Hughes et al., 2020 [42]	USA	39 Young adults (18-25	N= 39	Young adults with moderate-to-severe	Anxiety and Depressive	N/A	Program participants reported significantly

		years)		symptoms of depression and/or anxiety (n = 26) were enrolled in a 13-week intervention consisting of nutritional coaching and multivitamin supplements, weekly educational and peer support groups, and a modest financial stipend to engage with physical or expressive activities. A comparison group (n = 13) continued with their usual medication-based outpatient care.	symptoms		improved depression, anxiety, severity of distress, overall quality of life, and empowerment over 4 months, with progress maintained or further improved at 2-month follow-up. No evidence of change on any outcome was observed for comparison group participants.
Ibero-Baraibar et al., 2016 [43]	Spain	22 men and 25 women [mean ± SD age: 57 ± 5 y; body mass index (kg/m2): 30.6 ± 2.3]	N= 47	After a 1-wk run-in period, volunteers consumed 15% energy-restricted diets; one-half of the volunteers were randomly assigned to receive ready-to-eat meals supplemented with 1.4 g cocoa extract/d (645 mg total polyphenols/d), whereas the rest of	Anxiety and Depressive symptoms	Plasma monoamines [dopamine, dopac, and homovanillic acid (HVA)], monoamine oxidase (MAO), and psychological status (anxiety and depressive symptoms) were analyzed in fasting participants at baseline and endpoint.	Depressive symptoms decreased in both groups after the intervention (control: −9.4%, $P < 0.001$; cocoa: −6.3%, $P = 0.008$), but anxiety symptoms did not. A negative relation between changes in depressive symptoms and changes in plasma HVA was observed in the cocoa group ($\beta = -0.39$, $P = 0.029$).

				the volunteers received the same meals without cocoa supplementation.			
Inoue et al., 2018 [44]	Japan	38 participants (66-78 years)	N= 38	Over a 12-week period, 38 participants (66-78 years) underwent resistance training and were assigned to the probiotic Bifidobacterium supplementation (n=20; 1.25×1010 cfu each of Bifidobacteriumlongumsubsp.longum BB536, B.longum subsp.infantis M-63, Bifidobacteriumbreve M-16V and B.breve B-3) or the placebo (n=18) group.	Anxiety and Depressive symptoms	Japanese version of the Montreal Cognitive Assessment instrument (MoCA-J), Body mass index (BMI), Bristol Stool Form Scale (BSFS), Patient Health Questionnaire-9 (PHQ-9) Generalised Anxiety Disorder-7 (GAD-7), Mini Nutritional Assessment (MNA)	Only the probiotic group showed a significant decrease in the depression-anxiety scores (5.2±6.3 to 3.4±5.5, P=0.012)
Jackson et al., 2021 [45]	UK	56 healthy male and female individuals (18–54 years)	N= 56	Either a saffron extract or a placebo for 8 weeks.	Anxiety and Depressive symptoms	The POMS-2, STAI (Trait), the PSWQ, the PSS-10, the COPE Inventory (COPE), the Hospital Anxiety and Depression Scale (HADS), and the World Health Organization Quality of Life questionnaire	Participants who received the saffron extract reported reduced depression scores and improved social relationships at the end of the study. No evidence of an effect of saffron on subjective anxiety as assessed by the STAI questionnaire or anxiety

						(WHOQOL-BREF). The POMS "total mood disturbance" (TMD) score was the primary outcome measure.	scale of the HADS was found.
Jamilian et al., 2018 [46]	Iran	54 subjects, aged 18–40 years old.	N= 54	Subjects were randomly allocated to take either 1000 mg/d carnitine plus 200 μg/d chromium as chromium picolinate (*n* = 26) or placebo (*n* = 27) for 12 weeks.	Anxiety and Depressive symptoms	Beck Depression Inventory, General Health Questionnaire, Depression, Anxiety and Stress Scale	Carnitine and chromium co-supplementation, compared with the placebo, significantly improved beck depression inventory (*β* − 0.84; 95% CI, −1.51, −0.17; *p* = 0.01), general health questionnaire scores (*β* − 1.13; 95% CI, −2.13, −0.14; *p* = 0.02) and depression anxiety and stress scale scores (*β* − 0.96; 95% CI, −0.78, −0.14; *p* = 0.02).
Jamilian et al., 2018 [47]	Iran	60 subjects, aged 18–40 years old.	N= 60	Participants were randomly allocated into two groups to intake 8 × 10^9 CFU/day probiotic plus 200 μg/day selenium supplements (*n* = 30) or placebo (*n* = 30) for 12 weeks.	Anxiety and Depressive symptoms	Beck Depression Inventory, General Health Questionnaire, Depression, Anxiety and Stress Scale.	Probiotic and selenium co-supplementation resulted in a significant improvement in beck depression inventory (β − 0.76; 95% CI, − 1.26, − 0.26; *P* = 0.003), general health questionnaire scores (β − 1.15; 95% CI, − 1.97, − 0.32; *P* = 0.007) and depression anxiety and stress scale scores (β − 1.49; 95% CI, − 2.59,

							− 0.39; $P = 0.009$) compared with the placebo.
Jamilian et al., 2018 [48]	Iran	60 subjects, aged 18–40 years old with PCOS	N= 60	Subjects were randomly allocated to take either 50,000 IU vitamin D every 2 weeks plus 2000 mg/day omega-3 fatty acid from fish oil (*n* = 30) or placebo (*n* = 30) for 12 weeks.	Anxiety and Depressive Symptoms	Beck Depression Inventory, General Health Questionnaire, Depression, Anxiety and Stress Scale.	vitamin D and omega-3 fatty acid co-supplementation resulted in a significant improvement in beck depression inventory (−1.4 ± 1.6 vs. −0.5 ± 0.6, $P = 0.01$), general health questionnaire scores (−4.5 ± 4.3 vs. −1.9 ± 2.3, $P = 0.005$) and depression anxiety and stress scale scores (−5.0 ± 5.1 vs. −2.3 ± 3.5, $P = 0.01$) compared with the placebo.
Kaplan et al., 2015 [49]	Canada	Fifty-six adults aged 23–66 years	N= 56	A single nutrient (vitamin D, *n*=17), a few-nutrients formula (B-Complex, *n*=21), or a broad-spectrum mineral/vitamin formula (BSMV, *n*=18).	Anxiety and Depressive symptoms	Self-reported changes in depression, anxiety and stress were monitored for six weeks.	Although all groups showed substantial decreases on all measures, those consuming the B-Complex and the BSMV formulas showed significantly greater improvement in stress and anxiety compared with those consuming the single nutrient, with large effect sizes (Cohen's *d* range 0.76–1.08). There were no group differences between those consuming the B-Complex and BSMV.

Kiecolt-Glaser et al., 2011 [50]	USA	68 medical students	N= 68	The students received either *n*-3 (2.5 g/d, 2085 mg eicosapentaenoic acid and 348 mg docosahexanoic acid) or placebo capsules that mirrored the proportions of fatty acids in the typical American diet.	Anxiety and Depressive symptoms	(i)The Center for Epidemiological Studies Depression Scale (CES-D)<br>(ii)The Beck Anxiety Inventory<br>(iii)Women's Health Initiative Food Frequency Questionnaire (FFQ),<br>(iv)The Seven-Day Physical Activity Recall	Compared to controls, those students who received *n*-3 showed a 14% decrease in lipopolysaccharide (LPS) stimulated interleukin 6 (IL-6) production and a 20% reduction in anxiety symptoms, without significant change in depressive symptoms.
Kumar et al., 2011 [51]	India	54 adults	N= 54	bacosides enriched standardized extract of Bacopa monniera (BESEB-CDRI-08) (n=41) or an identical capsule placebo (n=13	Anxiety symptoms and insomnia	The effect on hemoglobin was determined by Sahli´s method, glucose estimation was done by semi auto analyzer, oxygen caring capacity and pulse by pulse oxy-meter, hand grip by muscle strength tester and anxiety, well-being, improvement in sleep abnormality and walking effect were evaluated by visual analog scale.	Treatment effect was significantly improved in anxiety, sleep abnormality and decrees in glucose level and period effect was significant in pulse. The findings suggest that BESEB-CDRI-08 significantly improves the rejuvenating effect by improving the above rejuvenating factor and thus confirms the rasayana effect.
Kyrou et al., 2017 [52]	Greece	36 participants (Females/Males: 31/5; age: 24.7±0.5 years)	N= 36	Two 4-week intervention periods (Melcalin hops or placebo; two 0.2 gr capsules once daily)	Anxiety and depressive symptoms	Anthropometric measurements, DASS-21 assessments and measurements of morning cortisol plasma	Significantly decreased DASS-21 anxiety, depression and stress scores were documented with hops (9.2±7.3 vs.

				separated by a 2-week wash-out		levels were performed at the beginning and the end of the 4-week treatment periods.	5.1±5.9, 11.9±7.9 vs. 9.2±7.4, and 19.1±8.1 vs. 11.6±8.1; all p values <0.05), which were significantly greater compared to those caused by the placebo (all p values <0.05).
Lewis et al., 2013 [53]	USA	60 adults diagnosed with major depression or other forms of depressive disorders.	N= 60	For the 60-day intervention period, participants received (a) Max Stress B (a whole nutrient natural source extract from probiotic colonies that contains vitamins B1, B2, B3, B5, B6, and B12, and folate, PABA, biotin, inositol, purified water, and certified organic alcohol) or (b) placebo (an oil/water emulsion with food coloring similar to the test product).	Anxiety and depressive symptoms	(i)The Beck Depression Inventory-II (BDI) (ii)Beck Anxiety Inventory (BAI) (iii)Medical Outcomes Study Short Form 36 (SF-36) (iv)Stanford 7-Day Physical Activity Recall	Max Stress B showed significant and more continuous improvements in depressive and anxiety symptoms, compared to placebo. Additionally, Max Stress B showed significant improvement on the mental health scale of the SF-36 compared to placebo.
Li et al., 2010 [54]	China	Three hundred and seventy-seven after-stroke elderly patients	N= 375	The 135 patients in the treated group were treated with Chinese medicine and general stroke-treatment, and the 126 patients in the control group were	Depressive, anxiety and insomnia symptoms	HAMD17 scores of neurological deficit (NDS), scores of sleep dysfunction rating scale (SDRS), Hamilton anxiety rating scale (HAMA), and treatment emergent symptom	The HAMD17, HAMA, SDRS, and NDS scores in the treated group after treatment were all improved more significantly than in the control group (P < 0.05).

				treated only with general stroke-treatment for 4 weeks.		scale (TESS) were observed before and after treatment.	
Lopresti et al., 2019 [55]	India	Sixty adults	N= 60	Either a placebo or 240 mg of a standardized ashwagandha extract (Shoden) once daily for 60 days	Anxiety and depressive symptoms	Outcomes were measured using the Hamilton Anxiety Rating Scale (HAM-A), Depression, Anxiety, and Stress Scale -21 (DASS-21), and hormonal changes in cortisol, dehydroepiandrosterone-sulphate (DHEA-S), and testosterone.	In comparison with the placebo, ashwagandha supplementation was associated with a statistically significant reduction in the HAM-A (P=.040) and a near-significant reduction in the DASS-21 (P=.096).
Macpherson et al., 2015 [56]	Australia	76 healthy women aged 50–75 years.	N= 76	Single multivitamin and mineral and herbal (MVMH) supplement versus placebo for 4 weeks	Anxiety and Depressive symptoms	Depression anxiety stress scale (DASS), state trait anxiety inventory–state anxiety scale and visual analogue scales (VAS).	It was demonstrated that the MVMH supplement improved overall DASS mood ratings; however, the most prominent effects appeared to be a reduction in ratings of perceived mental stress. These findings were confirmed using visual analogue scales, with these measures also demonstrating MVMH-related increased ratings of calmness. There were no benefits of the MVMH to mood ratings of depression and

							performance was not enhanced on the cognitive battery. Supplementation with a single multivitamin, mineral and herbal supplement reduces stress several hours after intake in healthy older people.
Mazidi et al., 2016 [57]	Iran	54 patients with anxiety and depression	N= 54	A 50 mg saffron capsule (Crocus sativus L. stigma) or a placebo capsule twice daily for 12 weeks.	Anxiety and Depressive Symptoms	Beck Depression Inventory (BDI) and Beck Anxiety Inventory (BAI) questionnaires were used	Saffron supplements had a significant effect on the BDI and BAI scores of subjects in comparison to placebo at the 12 week time-point ($p<0.001$).
Neri et al., 1995 [58]	Italy	60 subjects (18 M, 42 F), mean age 61 years, with age-associated memory impairment (AAMI)	N= 60	Standardized ginseng-containing vitamin complex or placebo for 9 months.	Anxiety and Depressive symptoms	Symptom Rating Test [SRT] (depression, anxiety, somatization, inadequacy) and The Randt Memory Test [RMT] (memory index [MI]), and Life Satisfaction in the Elderly Scale [LSES].	At final evaluation, SRT did not differ in the drug and placebo groups, whereas MI and LSES were significantly higher in the drug-treated group. Moreover, the negative correlation between the affective (SRT) and cognitive (MI) component of psychological well-being waned in the drug-treated but not placebo group.
Nakasone et al., 2015 [59]	Japan	54 adult subjects	N= 54	Capsule-form GE diet or placebo once daily for 8 weeks	Depressive symptoms and Sleep Quality	Profile of Mood States Short Form Japanese version II (POMS-S),	Supplementation with the GE diet appeared to be effective in improving various areas of perceived

						The Ogri-Shirakawa-Azumi sleep inventory MA version (OSA-MA) and Athens Insomnia Scale (AIS)	mood, as well as quantity and quality of sleep.
Noah et al., 2021 [60]	France	246 participants aged 18–50 years	N=246	magnesium + vitamin B6 combination (Magne B6®; 300 mg as magnesium lactate dihydrate and 30 mg vitamin B6 daily) or magnesium alone (Magnespasmyl®; 300 mg daily as magnesium lactate dihydrate) for 8 weeks	Depressive and anxiety symptoms	(i)DASS-42<br>(ii) QoL (Short Form-36 Health Survey)	Both treatment groups showed improved anxiety scores over the course of the 8-weeks study, with mean anxiety scores reducing from a severe level to a near normal level. Greatest improvement in mean anxiety score occurred in the first 4 weeks of treatment; the overall group mean score decreased from baseline by −5.86 (95% CI −6.67; −5.04). Across the overall population, participants in both treatment groups improved DASS-42 depression scores over the course of the 8-weeks study, such that depression scores reduced from moderate to normal (≤9). In both groups, most of the improvement in the depression score occurred in the first 4 weeks of

							treatment (mean decrease from baseline −5.54 [95% CI −6.47; −4.61 overall]).
Omori et al., 2017 [61]	Japan	29 Adult volunteers who were feeling fatigued and stressed daily	N=29	Amino acid-containing supplement and placebo were taken for 12 weeks	Depressive symptoms and sleep quality	The sleep status was evaluated using the Japanese version of the Pittsburgh Sleep Quality Index (PSQI-j). Fatigue and stress were evaluated using the Short form of Profile of Mood States (POMS-S).	The global score of PSQI-j after 12-week intake was significantly decreased with amino acid-containing supplement intake compared with that with placebo intake. On POMS-S, the measured values of anger-hostility (8th week) and the changes from pre-trial values of depression (4th week), anger-hostility (8th week), and confusion (12th week) showed significant improvement with amino acid-containing supplement intake compared with those with placebo intake
Ostadmohammadi et al., 2021 [62]	Iran	60 subjects, aged 18-40 years old.	N=60	Either 50,000 IU vitamin D every 2 weeks plus 8 × 10^9 CFU/day probiotic (n = 30) or placebo (n = 30) for 12 weeks.	Anxiety and depressive symptoms	Depression, Anxiety Stress Scale-21, Beck Depression Inventory, General Health Questionnaire	Vitamin D and probiotic co-supplementation, compared with the placebo, significantly improved beck depression inventory [β (difference in the mean of outcomes measures between treatment groups) - 0.58; 95% CI, - 1.15, - 0.02; P = 0.04], general health questionnaire scores (β -

							0.93; 95% CI, - 1.78, - 0.08; P = 0.03) and depression, anxiety and stress scale scores (β - 0.90; 95% CI, - 1.67, - 0.13; P = 0.02).
Pouteau et al., 2018 [63]	France	264 subjects	N= 249	Magnesium–vitamin B6 combination (Magne B6 [Mg–vitamin B6]; daily dose 300 mg and 30 mg, respectively) or magnesium alone (Magnespasmyl [Mg]; daily dose 300 mg)	Anxiety and depressive symptoms	DASS-42	In the modified intention-to-treat analysis (N = 264 subjects), both treatment arms substantially reduced DASS-42 stress subscale score from baseline to Week 8 (Mg–vitamin B6, 44.9%; Mg 42.4%); no statistical difference between arms was observed (p>0.05).
Raygan et al., 2018 [64]	Iran	60 diabetic people with Coronary Heart Disease, aged 45–85 years old.	N=60	Either 50,000 IU vitamin D every 2 weeks plus 8×10^9 CFU/g probiotic of Lactocare Zisttakhmir Co (n = 30) or placebo (n = 30) for 12 weeks.	Anxiety and depressive symptoms	Beck Depression Inventory, Beck Anxiety Inventory and General Health Questionnaire	After the 12-week intervention, compared with the placebo, vitamin D and probiotic supplementation resulted in significant improvements in beck depression inventory
Remenapp et al., 2022 [65]	USA	60 Healthy adults (*n* = 43 women and *n* = 17 men; mean age = 34.41 years)	N=60	Ashwagandha (400 mg/d), Ashwagandha (225 mg/d), and placebo for 30 days.	Anxiety and depressive symptoms	CNS vital signs, self-reported measures of Trait Anxiety Inventory, Depression Anxiety Stress Scale, Perceived Stress Scale, and Food	For the self-report assessments, significant main effects for time were evidenced for anxiety, depression, perceived stress, and food cravings, *p*'s < 0.01. The

						Cravings Questionnaire-15.	main effect for group and the interactions were non-significant.
Roca et al., 2016 [66]	Netherlands, Spain, UK, Germany	1000 participants aged 18–75 years with body mass index between 25–40 kg/m^2 and with a Patient Health Questionnaire -9 score ≥ 5	N=1000	Daily multi-nutrient supplement (omega-3 fatty acids, calcium, selenium, B-11 vitamin and D-3 vitamin) versus placebo, and/or FBC therapy sessions versus usual care. Interventions last 12 months.	Depressive and anxiety symptoms	Primary endpoint is the onset of an episode of MDD, assessed according to DSM-IV based criteria using the MINI 5.0 interview. Depressive symptoms, anxiety, food and eating behavior, physical activity and health related quality of life are secondary outcomes.	The trial aims to provide a better understanding of the causal role of specific nutrients, overall diet, and food-related behavior change with respect to the incidence of MDD episodes. This knowledge will be used to develop and disseminate innovative evidence-based, feasible, and effective nutritional public health strategies for the prevention of clinical depression.
Saccarello et al., 2020 [67]	Italy	90 participants	N= 90	SAMe plus *L. plantarum* HEAL9 (n = 46) or placebo (n = 44)	Sleep Quality, Anxiety and depressive symptoms	Zung Self-Rating Depression Scale (Z-SDS), Zung Self-Rating Anxiety Scale (Z-SAS), Insomnia Severity Index (ISI),Birmingham IBS symptom questionnaire (B-IBS), and EQ-5D-3L	A greater reduction for the new combination compared to placebo was seen at treatment week 6 in the Z-SDS total score (*P* = .0165) and the core depression subdomain (*P* = .0247). A significant reduction in favor of the combination was shown at treatment week 2 for the Z-SDS total score (*P* = .0330), the cognitive and anxiety subdomains (*P* = .0133 and *P* = .0459, respectively),

							and the anxiety questionnaire (*P* = .0345). No treatment-related adverse events occurred.
Salleh et al., 2021 [68]	Malaysia	Thirty university badminton players aged from 19 to 22 years old	N= 30	Two groups, where the probiotic group (PG; n = 15) received a drink that contained *Lactobacillus casei* Shirota (3 × 10^{10} CFU) and placebo group (CG; n = 15) a placebo drink for six weeks.	Anxiety and depressive symptoms	Anxiety, stress and mood levels were determined using the revised competitive state anxiety inventory-2 (CSAI-2R), Perceived Stress Scale and Brunel Mood Scale questionnaires, respectively.	After six weeks, the anxiety and stress levels of PG players significantly decreased by 16% ($p < 0.001$) and 20% ($p < 0.001$), respectively, but there were no significant changes detected in CG players.
Salve et al., 2019 [69]	India	58 male and female participants	N= 58	Capsules of Ashwagandha extract 125 mg, Ashwagandha extract 300 mg or identical placebo twice daily for eight weeks in a 1:1:1 ratio.	Anxiety symptoms and sleep quality	Perceived Stress Scale, Hamilton-Anxiety (HAM-A) scale and seven-point sleep scale.	A significant reduction in PSS scores was observed with Ashwagandha 250 mg/day (P < 0.05) and 600 mg/day (P < 0.001). Serum cortisol levels reduced with both Ashwagandha 250 mg/day (P < 0.05) and Ashwagandha 600 mg/day (P < 0.0001). Compared to the placebo group participants, the participants receiving Ashwagandha had significant improvement in sleep quality. The results show that Ashwagandha 600 mg/day treatment is

							effective in reducing anxiety
Sanders et al., 2011 [70]	Australia	118 participants	N= 118	500 000 IU vitamin D_3 (cholecalciferol) orally or placebo every autumn/winter for 3–5 consecutive years.	Anxiety and depressive symptoms	General Health Questionnaire, the 12-item Short Form Health Survey, the Patient Global Impression–Improvement scale and the WHO Well-Being Index.	In this non-clinical population, no significant differences between the vitamin D and placebo groups were detected in any of the measured outcomes of mental health. Serum 25-hydroxyvitamin D levels in the vitamin D group were 41% higher than the placebo group 12 months following their annual dose. Despite this difference, scores from the questionnaires did not differ. Furthermore, there was no interaction between those on antidepressant/anxiety medication at baseline and the treatment groups.
Schaafsma et al., 2021 [71]	Netherland s	70 adults with sleep disturbances	N= 70	A dairy-based product (DP) containing protein, galacto-oligosaccharides, vitamins and minerals was consumed1 h before bed-time (three weeks intervention;	Depressive symptoms, anxiety and sleep quality	Pittsburgh Sleep Quality Index (PSQI), Depression Anxiety Stress Scale (DASS-42)	Fecal samples were collected in the 1st intervention period only. Compared to placebo (skimmed milk), PSQI was only lower at day 14 in the 2nd intervention period in intention-to-treat (ITT) (p = 0.017; n = 69) and per-protocol (PP) (p = 0.038; n =

				three weeks washout)			64) analyses. Post-hoc analysis (modified-PP: n=47, with baseline PSQI ≥ 9, and endline day 14), however, showed a decrease in PSQI (−1.60 ± 2.53; p = 0.034). No differences were found for total DASS-42 score, or any of the sub-scores, between DP and placebo in ITT, PP, or modPP populations.
Scholey et al., 2017 [72]	Australia	171 participants	N= 171	Daily treatment for 2 weeks with LZComplex3 or placebo.	Depressive symptoms, anxiety and sleep quality	Pittsburgh Sleep Quality Index (PSQI), State-Trait Anxiety Inventory (STAI) State subscale (STAI-S), Leeds Sleep Evaluation Questionnaire (LSEQ) , Epworth Sleepiness Scale (ESS), Insomnia Severity Index (ISI) , Consensus Sleep Diary (CSD) and Hospital Anxiety and Depression Scale (HADS)	Results revealed that sleep quality measured by change in Pittsburgh Sleep Quality Index (PSQI) score improved in both the LZComplex3 and placebo groups. There were no significant between group differences between baseline and endpoint on the primary outcome. The majority of secondary outcomes, which included daytime functioning and physical fatigue, mood and anxiety, cognitive performance, and stress reactivity, showed similar improvements in the LZComplex3 and placebo groups.

Shabani et al.,2019 [73]	Iran	58 subjects, aged 18–40 years old.	N= 58	Subjects were randomly allocated to take either 10 mg melatonin (2 melatonin capsules, 5 mg each) ($n = 29$) or placebo ($n = 29$) once a day 1 h before bedtime for 12 weeks.	Depressive symptoms, anxiety and sleep quality	Pittsburgh Sleep Quality Index, Beck Depression Inventory index and Beck Anxiety Inventory index	Melatonin supplementation significantly decreased Pittsburgh Sleep Quality Index (β −2.15; 95% CI, −3.62, −0.68; $P = 0.005$), Beck Depression Inventory index (β −3.62; 95% CI, −5.53, −1.78; $P<0.001$) and Beck Anxiety Inventory index (β −1.95; 95% CI, −3.41, −0.48; $P = 0.01$) compared with the placebo.
Shetty et al., 2021 [74]	India	198 eligible participants (140 female an d 58 male)	N= 198	Two 500 mg capsule of Brahmi (Bacopa monnieri (L.) Pennell) was administered twice daily after food in morning and night for 30 days.	Anxiety and Depressive symptoms	Depression Anxiety and Stress Scale-21 (DASS-21)	At the DASS-21, 4.09% of the responders presented pathological levels of depression, 10.85% of anxiety, and 0.72% of stress among general population. Significant decrease in means were found for scales of depression, an xiety, stress and total DASS-21 after intervention ($p<0.001$) with Brahmi for a period of 1 month
Sim et al., 2022 [75]	South Korea	46 healthy young adults (20–39 years)	N= 46	500 mg of vitamin C twice a day for 4 weeks ($n = 24$) or a placebo ($n = 22$).	Anxiety and Depressive symptoms	Stress Response Inventory (SRI), Beck Depression Inventory (BDI), State Trait Anxiety Inventory (STAI) and Positive and	No significant effects of vitamin C were seen on stress, depression, positive and negative affect, or state anxiety (all $p > 0.05$)

						Negative Affect Schedule (PANAS)	
Slow et al., 2014 [76]	New Zealand	308 healthy adults (241 women; 81 men) aged 18-67	N= 308	Participants were randomized to receive an oral dose of either 200 000 IU vitamin D_3 monthly for two months then 100 000 IU monthly (n=161) or placebo (n=161) for a total of 18 months.	Sleep quality, Anxiety and Depressive symptoms	The earthquake impact questionnaire	There was no significant difference in the number of self-reported adverse effects between those receiving vitamin D supplementation and those receiving placebo. There was also no difference in the overall adverse impact score between treatment groups (χ^2 P=0.44). The exception was that those in the vitamin D group experienced more adverse effects on family relationships (22% *v* 13%; χ^2 P=0.03). The number of psychological adverse events—such as fatigue, stress, anxiety, and insomnia—that participants reported at their usual monthly appointments was significantly higher after the earthquake (χ^2 P=0.007) but did not differ between treatment groups.

Table S6. Synthetic summary of the main findings of each category of studies included in this systematic review (e.g., per health outcome), in addition to their implications for public health policy and clinical practice.

Health outcome category	Summary of main findings	Implications of findings
Anxiety symptoms	While some supplements did not show any significant benefits, others, such as L-Lysine HCI and L-arginine capsules and a multivitamin supplement containing high levels of B-vitamins, were found to significantly reduce anxiety symptoms in healthy participants. Questionnaires were the primary tool used to measure anxiety symptoms, although some studies used objective measures such as heart rate variability and salivary IgA.	The findings suggest that certain plant-based and nutritional supplements may have potential benefits for reducing anxiety symptoms. However, further research is needed to determine the efficacy of these supplements, and clinical guidelines should be cautious in recommending them without sufficient evidence.
Depressive symptoms	The relationship between vitamin and nutrient intake and depressive symptoms remains mixed in the literature. While some studies did not find significant associations between vitamin D3, Vitamin C, and folate supplementation and depressive symptoms, others found that increasing intake of multiple individual nutrients, fruits, and vegetables, receiving vitamin D supplementation, and taking multivitamin supplements were associated with improved depressive symptoms. B vitamins may also be promising for treating and managing Major Depressive Disorders.	It is important to consider the mixed results of studies when determining the best interventions for anxiety and depression, but some promising options include supplements containing L-Lysine HCI and L-arginine, B-vitamins, and synbiotics. Increasing intake of multiple individual nutrients, fruits, and vegetables may also improve depressive symptoms. Public health interventions could include promoting healthy eating habits, educating individuals on the potential benefits of supplements, and providing access to mental health resources.
Sleep health outcomes	Several supplements, including natural melatonin, saffron, L-ornithine, and vitamin D, were associated with better sleep quality in six studies. However, Ayurveda did not have an effect on sleep quality, and in some cases, supplements improved sleep due to their effect on an underlying medical condition.	The findings suggest that some supplements may be effective in improving sleep quality, such as natural melatonin, saffron, L-ornithine, and vitamin D. However, interventions targeting underlying medical conditions may also improve sleep quality. Healthcare providers can consider recommending specific supplements or addressing underlying medical conditions to improve sleep quality in their patients. It is important to note that supplementation should be approached with caution and under the guidance of a healthcare professional.
	Some studies found that vitamin D supplementation combined with omega-3 fatty acid or probiotics significantly improved depressive symptoms, while other studies found that multivitamin formulations or probiotics supplementation alone were associated with improvements in anxiety symptoms. However, there remains evidence in the literature that found no significant effect of vitamin D supplementation on anxiety or depressive symptoms.	The findings suggest that combining vitamin D supplementation with omega-3 fatty acids or probiotics may be effective in improving depressive symptoms, and multivitamin formulations or probiotic supplementation alone may be helpful for anxiety symptoms. Clinicians may consider recommending these supplements as part of a comprehensive treatment plan for individuals with anxiety or depressive symptoms, but patients should always consult with their healthcare provider before starting any new supplements.

Table S7. Overall risk-of-bias assessment for studies included in this review, presented for different domains according to the RoB2 risk assessment tool.

	Selection Bias		Performance Bias	Detection Bias	Attrition Bias	Reporting Bias	Other Bias	
**Article**	**Randomized selection in the population**	**Adequate concealment of allocation to intervention**	**Double Blinding**	**Blinding of Outcome Assessment**	**Validated completeness of outcome data**	**Adequate selective outcome reporting**	**Report of drop out during follow up**	**Clear definition of inclusion/exclusion criteria**
Giles et al., 2015 [1]	-	+	+	-	+	+	+	+
Gonzalez et al., 2018 [2]	+	+	+	+	+	+	+	+
Hadi et al., 2019 [3]	+	+	+	-	+	+	+	+
Jaatinen et al., 2014 [4]	+	+	+	+	+	+	+	+
Noorwali et al., 2016 [5]	+	?	+	+	+	+	-	+
Pipingas et al., 2013 [6]	-	?	+	+	+	+	+	+
Smriga et al., 2007 [7]	+	+	+	-	-	+	+	+
Ghorbani et al., 2018 [8]	+	+	+	+	+	+	+	+
Gosney et al., 2008 [9]	+	+	+	+	?	+	+	+
Imaoka et al., 2019 [10]	+	-	-	-	+	+	+	+
Jorde et al., 2008 [11]	+	+	+	+	-	+	+	+
Kaviani et al., 2020 [12]	+	+	+	+	+	+	+	+
Kenny et al., 2004	+	?	-	-	+	+	+	+
Kazemi et al., 2019 [13]	+	+	+	+	+	+	+	+
Krikorian et al., 2013 [14]	+	+	+	?	+	+	?	+
Lee et al., 2008 [15]	+	?	+	?	?	+	?	+
Lee et al., 2016 [16]	-	-	-	-	+	+	+	?
Lespérance et al., 2010 [17]	+	?	+	?	?	?	+	+
Lucas et al., 2008 [18]	+	+	+	+	+	+	+	+
Mech & Farah, 2016 [19]	+	?	+	?	+	+	+	+

Moore et al., 2018 [20]	+	?	+	?	+	+	?	+
Ng et al., 2016 [21]	+	+	-	-	+	+	+	+
Okereke et al., 2020 [23]	+	?	+	+	+	+	+	+
Parletta et al., 2017 [24]	+	+	-	-	-	+	+	+
Rice et al., 2014 [25]	+	+	+	+	+	?	?	+
Rizzo et al., 2012 [26]	+	+	+	+	+	+	?	+
Rondanelli et al., 2011 [27]	+	+	+	+	?	+	?	+
Moghaddam et al.,2017 [29]	+	+	-	-	+	+	+	+
Shinto et al., 2016 [30]	+	+	+	+	-	+	-	+
Gandolfi et al., 2020 [31]	+	-	+	+	+	+	+	+
Gong et al., 2021 [32]	+	+	+	+	+	+	+	+
Lopresti et al., 2021 [33]	+	+	+	+	+	+	+	+
Manjunath & Telles, 2005 [34]	+	+	-	-	+	+	+	+
Miyake et al., 2014 [35]	+	+	+	?	+	+	+	+
Majid et al., 2017 [36]	+	+	+	+	+	+	+	+
Kim et al., 2021 [37]	+	+	+	+	+	+	+	+
Koning et al., 2019 [38]	+	+	+	+	+	+	+	+
Harris et al., 2011 [39]	+	+	+	+	+	+	+	+
Hegde et al., 2020 [40]	+	+	-	-	+	+	-	+
Hoffman et al., 2020 [41]	+	?	+	+	+	+	+	?
Hughes et al., 2020 [42]	-	-	-	-	+	+	+	+
Ibero-Baraibar et al., 2016 [43]	+	+	-	-	?	+	+	+
Inoue et al., 2018 [44]	+	+	+	?	+	+	+	+
Jackson et al., 2021 [45]	+	+	+	+	+	+	+	+
Jamilian et al., 2018 [46]	+	+	+	+	+	+	+	+
Jamilian et al., 2018 [47]	+	?	+	+	+	+	+	+

Jamilian et al., 2018 [48]	+	+	+	+	+	+	+	+
Kaplan et al., 2015 [49]	+	-	-	-	+	+	+	+
Kiecolt-Glaser et al., 2011 [50]	+	+	+	+	+	+	+	+
Kumar et al., 2011 [51]	+	+	+	+	+	+	+	+
Kyrou et al., 2017 [52]	+	+	+	+	+	+	+	+
Lewis et al., 2013 [53]	+	+	+	+	+	+	+	+
Li et al., 2010 [54]	+	?	?	?	?	+	?	?
Lopresti et al., 2019 [55]	+	+	+	+	+	+	+	+
Macpherson et al., 2015 [56]	+	?	+	+	+	+	+	+
Mazidi et al., 2016 [57]	+	+	+	+	+	+	?	+
Neri et al., 1995 [58]	+	?	+	?	+	+	+	+
Nakasone et al., 2015 [59]	+	?	+	?	?	+	?	?
Noah et al., 2021 [60]	+	+	-	?	+	+	+	+
Omori et al., 2017 [61]	+	?	+	?	?	+	?	?
Ostadmohammadi et al., 2021 [62]	+	+	+	+	+	+	+	+
Pouteau et al., 2018 [63]	+	+	-	-	+	+	+	+
Raygan et al., 2018 [64]	+	+	+	+	+	+	+	+
Remenapp et al., 2022 [65]	+	?	+	+	+	+	+	+
Roca et al., 2016 [66]	+	+	-	?	+	+	+	+
Saccarello et al., 2020 [67]	+	+	+	+	+	+	+	+
Salleh et al., 2021 [68]	+	?	+	+	+	+	+	+
Salve et al., 2019 [69]	+	+	+	+	+	+	+	+
Sanders et al., 2011 [70]	+	+	+	+	+	+	+	+
Schaafsma et al., 2021 [71]	+	+	+	+	-	+	-	?

Scholey et al., 2017 [72]	+	+	+	?	+	+	+	+
Shabani et al.,2019 [73]	+	+	+	+	+	+	+	+
Shetty et al., 2021 [74]	+	?	-	-	?	?	?	?
Sim et al., 2022 [75]	+	+	+	+	+	+	+	+
Slow et al., 2014 [76]	+	+	+	+	+	+	+	+

+ = Low risk of bias

? = Some risk of bias

- = High risk of bias

References

1. Giles, G.E.; Mahoney, C.R.; Urry, H.L.; Brunyé, T.T.; Taylor, H.A.; Kanarek, R.B. Omega-3 fatty acids and stress-induced changes to mood and cognition in healthy individuals. *Pharmacol. Biochem. Behav.* **2015**, *132*, 10–19. https://doi.org/10.1016/j.pbb.2015.02.018.
2. Gonzalez, A.M.; Sell, K.M.; Ghigiarelli, J.J.; Spitz, R.W.; Accetta, M.R.; Mangine, G.T. Effect of Multi-Ingredient Supplement Containing Satiereal, Naringin, and Vitamin D on Body Composition, Mood, and Satiety in Overweight Adults. *J. Diet. Suppl.* **2018**, *15*, 965–976. https://doi.org/10.1080/19390211.2017.1407385.
3. Hadi, A.; Sepandi, M.; Marx, W.; Moradi, S.; Parastouei, K. Clinical and psychological responses to synbiotic supplementation in obese or overweight adults: A randomized clinical trial. *Complement. Ther. Med.* **2019**, *47*, 102216. https://doi.org/10.1016/j.ctim.2019.102216.
4. Jaatinen, N.; Korpela, R.; Poussa, T.; Turpeinen, A.; Mustonen, S.; Merilahti, J.; Peuhkuri, K. Effects of daily intake of yoghurt enriched with bioactive components on chronic stress responses: A double-blinded randomized controlled trial. *Int. J. Food Sci. Nutr.* **2014**, *65*, 507–514. https://doi.org/10.3109/09637486.2014.880669.
5. Noorwali, E.A.; Beaumont, J.D.; Corfe, B.M.; Owen, L. The effects of probiotic supplementation on emotional memory and pain response. *Proc. Nutr. Soc.* **2017**, *76*, E6. https://doi.org/10.1017/S0029665117000064.
6. Pipingas, A.; Camfield, D.A.; Stough, C.; Cox, K.H.M.; Fogg, E.; Tiplady, B.; Sarris, J.; White, D.J.; Sali, A.; Wetherell, M.A.; et al. The effects of multivitamin supplementation on mood and general well-being in healthy young adults. A laboratory and at-home mobile phone assessment. *Appetite* **2013**, *69*, 123–136. https://doi.org/10.1016/j.appet.2013.05.016.
7. Smriga, M.; Ando, T.; Akutsu, M.; Furukawa, Y.; Miwa, K.; Morinaga, Y. Oral treatment with L-lysine and L-arginine reduces anxiety and basal cortisol levels in healthy humans. *Biomed. Res.* **2007**, *28*, 85–90. https://doi.org/10.2220/biomedres.28.85.
8. Ghorbani, Z.; Nazari, S.; Etesam, F.; Nourimajd, S.; Ahmadpanah, M.; Jahromi, S.R. The effect of synbiotic as an adjuvant therapy to fluoxetine in moderate depression: A randomized multicenter trial. *Arch. Neurosci.* **2018**, *5*. https://doi.org/10.5812/archneurosci.60507.
9. Gosney, M.A.; Hammond, M.F.; Shenkin, A.; Allsup, S. Effect of micronutrient supplementation on mood in nursing home residents. *Gerontology* **2008**, *54*, 292–299. https://doi.org/10.1159/000131886.
10. Imaoka, M.; Nakao, H.; Nakamura, M.; Tazaki, F.; Maebuchi, M.; Ibuki, M.; Takeda, M. Effect of Multicomponent Exercise and Nutrition Support on the Cognitive Function of Older Adults: A Randomized Controlled Trial. *Clin. Interv. Aging* **2019**, *14*, 2145–2153. https://doi.org/10.2147/CIA.S229034.
11. Jorde, R.; Sneve, M.; Figenschau, Y.; Svartberg, J.; Waterloo, K.; Jorde, R.; Sneve, M.; Figenschau, Y.; Svartberg, J.; Waterloo, K. Effects of vitamin D supplementation on symptoms of depression in overweight and obese subjects: Randomized double blind trial. *J. Intern. Med.* **2008**, *264*, 599–609. https://doi.org/10.1111/j.1365-2796.2008.02008.x.
12. Kaviani, M.; Nikooyeh, B.; Zand, H.; Yaghmaei, P.; Neyestani, T.R. Effects of vitamin D supplementation on depression and some involved neurotransmitters. *J. Affect. Disord.* **2020**, *269*, 28–35. https://doi.org/10.1016/j.jad.2020.03.029.
13. Kazemi, A.; Noorbala, A.A.; Azam, K.; Eskandari, M.H.; Djafarian, K. Effect of probiotic and prebiotic vs placebo on psychological outcomes in patients with major depressive disorder: A randomized clinical trial. *Clin. Nutr.* **2019**, *38*, 522–528. https://doi.org/10.1016/j.clnu.2018.04.010.
14. Krikorian, R.; Eliassen, J.C.; Boespflug, E.L.; Nash, T.A.; Shidler, M.D. Improved cognitive-cerebral function in older adults with chromium supplementation. *Nutr. Neurosci.* **2010**, *13*, 116–122. https://doi.org/10.1179/147683010X12611460764084.
15. Lee, D.M.; Tajar, A.; Ulubaev, A.; Pendleton, N.; O'Neill, T.W.; O'Connor, D.B.; Bartfai, G.; Boonen, S.; Bouillon, R.; Casanueva, F.F.; et al. Association between 25-hydroxyvitamin D levels and cognitive performance in middle-aged and older European men. *J. Neurol. Neurosurg. Psychiatry* **2009**, *80*, 722–729. https://doi.org/10.1136/jnnp.2008.165720.
16. Lee, H.K.; Kim, S.Y.; Sok, S.R. Effects of Multivitamin Supplements on Cognitive Function, Serum Homocysteine Level, and Depression of Korean Older Adults With Mild Cognitive Impairment in Care Facilities. *J. Nurs. Scholarsh.* **2016**, *48*, 223–231. https://doi.org/10.1111/jnu.12201.
17. Lespérance, F.; Frasure-Smith, N.; St-André, E.; Turecki, G.; Lespérance, P.; Wisniewski, S.R. The efficacy of omega-3 supplementation for major depression: A randomized controlled trial. *J. Clin. Psychiatry* **2011**, *72*, 1054–1062. https://doi.org/10.4088/JCP.10m05966blu.

18. Lucas, M.; Asselin, G.; Mérette, C.; Poulin, M.J.; Dodin, S. Ethyl-eicosapentaenoic acid for the treatment of psychological distress and depressive symptoms in middle-aged women: A double-blind, placebo-controlled, randomized clinical trial. *Am. J. Clin. Nutr.* **2009**, *89*, 641–651. https://doi.org/10.3945/ajcn.2008.26749.
19. Mech, A.W.; Farah, B.A. Reduced B vitamin therapy for MDD in MTHFR C677t/A1298c patients-clinical response correlates with homocysteine reduction: A double-blind, placebo-controlled study. *CNS Spectr.* **2016**, *21*, 116–117. https://doi.org/10.1017/S1092852915000905.
20. Moore, K.; Porter, K.; Doherty, L.C.; Hughes, C.F.; Ward, M.; Hoey, L.; Strain, J.J.; Pentieva, K.; McNulty, H. A randomised controlled trial of B-vitamin supplementation on neuropsychiatric performance: Results from the BrainHOP trial. *Proc. Nutr. Soc.* **2018**, *77*, E73. https://doi.org/10.1017/S0029665118000770.
21. Ng, T.-P.; Chan, G.; Nyunt, M.; Feng, L.; Niti, M.; Tan, B.; Khoo, S.; Chan, S.; Yap, P.; Yap, K. Multi-domains lifestyle interventions reduces depressive symptoms among frail and pre-frail older persons: Randomized controlled trial. *J. Nutr. Health Aging* **2017**, *21*, 918–926. https://doi.org/10.1007/s12603-016-0867-y.
22. Nguyen, H.D.; Oh, H.; Kim, M.-S. Higher intakes of nutrients are linked with a lower risk of cardiovascular diseases, type 2 diabetes mellitus, arthritis, and depression among Korean adults. *Nutr. Res.* **2022**, *100*, 19–32. https://doi.org/10.1016/j.nutres.2021.11.003.
23. Okereke, O.I.; Reynolds Iii, C.F.; Mischoulon, D.; Chang, G.; Vyas, C.M.; Cook, N.R.; Weinberg, A.; Bubes, V.; Copeland, T.; Friedenberg, G.; et al. Effect of Long-term Vitamin D3 Supplementation vs Placebo on Risk of Depression or Clinically Relevant Depressive Symptoms and on Change in Mood Scores: A Randomized Clinical Trial. *JAMA J. Am. Med. Assoc.* **2020**, *324*, 471–480. https://doi.org/10.1001/jama.2020.10224.
24. Parletta, N.; Zarnowiecki, D.; Cho, J.; Wilson, A.; Bogomolova, S.; Villani, A.; Itsiopoulos, C.; Meyer, B.; Segal, L.; O'Dea, K. Effects of a Mediterranean-style diet supplemented with fish oil on mental health and quality of life in people with depression. *Nutr. Neurosci.* **2018**, *21*, S41. https://doi.org/10.1080/1028415X.2018.1449784.
25. Rice, S.M.; Hickie, I.B.; Yung, A.R.; Mackinnon, A.; Berk, M.; Davey, C.; Hermens, D.F.; Hetrick, S.E.; Parker, A.G.; Schäfer, M.R.; et al. Youth depression alleviation: The Fish Oil Youth Depression Study (YoDA-F): A randomized, double-blind, placebo-controlled treatment trial. *Early Interv. Psychiatry* **2016**, *10*, 290–299. https://doi.org/10.1111/eip.12166.
26. Rizzo, A.M.; Corsetto, P.A.; Montorfano, G.; Opizzi, A.; Faliva, M.; Giacosa, A.; Ricevuti, G.; Pelucchi, C.; Berra, B.; Rondanelli, M. Comparison between the AA/EPA ratio in depressed and non depressed elderly females: Omega-3 fatty acid supplementation correlates with improved symptoms but does not change immunological parameters. *Nutr. J.* **2012**, *11*, 82–82. https://doi.org/10.1186/1475-2891-11-82.
27. Rondanelli, M.; Giacosa, A.; Opizzi, A.; Pelucchi, C.; La Vecchia, C.; Montorfano, G.; Negroni, M.; Berra, B.; Politi, P.; Rizzo, A.M. Long chain omega 3 polyunsaturated fatty acids supplementation in the treatment of elderly depression: Effects on depressive symptoms, on phospholipids fatty acids profile and on health-related quality of life. *J. Nutr. Health Aging* **2011**, *15*, 37–44. https://doi.org/10.1007/s12603-011-0011-y.
28. Rondanelli, M.; Opizzi, A.; Antoniello, N.; Boschi, F.; Iadarola, P.; Pasini, E.; Aquilani, R.; Dioguardi, F.S. Effect of essential amino acid supplementation on quality of life, Amino acid profile and strength in institutionalized elderly patients. *Clin. Nutr.* **2011**, *30*, 571–577. https://doi.org/10.1016/j.clnu.2011.04.005.
29. Shapouri-Moghaddam, A.; Bagherniya, M.; Ehteshamfar, S.M.; Rahimi, H.; Safarian, M. High fish consumption decreased the likelihood of depressive symptoms in community-living older people: A randomized-controlled trial. *J. Gerontol. Geriatr.* **2017**, *65*, 232–237.
30. Shinto, L.; Marracci, G.; Mohr, D.C.; Bumgarner, L.; Murchison, C.; Senders, A.; Bourdette, D. Omega-3 Fatty Acids for Depression in Multiple Sclerosis: A Randomized Pilot Study. *PloS ONE* **2016**, *11*, e0147195. https://doi.org/10.1371/journal.pone.0147195.
31. Gandolfi, J.V.; Bernardo, A.P.A.D.; Chanes, D.A.V.; Martin, D.F.; Joles, V.B.; Amendola, C.P.; Sanches, L.C.; Ciorlia, G.L.; Lobo, S.M. The Effects of Melatonin Supplementation on Sleep Quality and Assessment of the Serum Melatonin in ICU Patients: A Randomized Controlled Trial. *Crit. Care Med.* **2020**, *48*, E1286–E1293. https://doi.org/10.1097/CCM.0000000000004690.
32. Gong, Y.-C.; Chen, X.; Song, Q.-T.; Gan, Y.; Zhang, B.; Li, B.-S.; Chen, Z.; He, Y. A randomized placebo-controlled study: Phellodendron Bawei tablets combined with standard management can improve storage symptoms, sleep quality, and medication compliance in patients with benign prostatic hyperplasia compared to placebo with standard management. *Transl. Androl. Urol.* **2021**, *10*, 3423–3431. https://doi.org/10.21037/tau-21-588.
33. Lopresti, A.L.; Smith, S.J.; Drummond, P.D. An investigation into an evening intake of a saffron extract (affron®) on sleep quality, cortisol, and melatonin concentrations in adults with poor sleep: A randomised, double-blind, placebo-controlled, multi-dose study. *Sleep Med.* **2021**, *86*, 7–18. https://doi.org/10.1016/j.sleep.2021.08.001.

34. Manjunath, N.K.; Telles, S. Influence of Yoga and Ayurveda on self-rated sleep in a geriatric population. *Indian J. Med. Res.* **2005**, *121*, 683–690.
35. Miyake, M.; Kirisako, T.; Kokubo, T.; Miura, Y.; Morishita, K.; Okamura, H.; Tsuda, A. Randomised controlled trial of the effects of L-ornithine on stress markers and sleep quality in healthy workers. *Nutr. J.* **2014**, *13*, 53–53. https://doi.org/10.1186/1475-2891-13-53.
36. Majid, M.S.; Ahmad, H.S.; Bizhan, H.; Hosein, H.Z.M.; Mohammad, A. The effect of vitamin D supplement on the score and quality of sleep in 20–50 year-old people with sleep disorders compared with control group. *Nutr. Neurosci.* **2018**, *21*, 511–519.
37. Kim, M.; Choi, E.-J.; Kwon, O.J.; Park, H.-J.; Kim, A.-R.; Seo, B.-N.; Chung, S.-Y.; Lee, J.-H.; Kim, J.-H. Electroacupuncture plus moxibustion for major depressive disorder: A randomized, sham-controlled, pilot clinical trial. *Integr. Med. Res.* **2021**, *10*, 100727. https://doi.org/10.1016/j.imr.2021.100727.
38. Koning, E.J.d.; Lips, P.; Penninx, B.W.J.H.; Elders, P.J.M.; Heijboer, A.C.; Heijer, M.d.; Bet, P.M.; Marwijk, H.W.J.v.; Schoor, N.M.v. Vitamin D supplementation for the prevention of depression and poor physical function in older persons: The D-Vitaal study, a randomized clinical trial. *Am. J. Clin. Nutr.* **2019**, *110*, 1119–1130. https://doi.org/10.1093/ajcn/nqz141.
39. Harris, E.; Kirk, J.; Rowsell, R.; Vitetta, L.; Sali, A.; Scholey, A.B.; Pipingas, A. The effect of multivitamin supplementation on mood and stress in healthy older men. *Hum. Psychopharmacol.* **2011**, *26*, 560–567. https://doi.org/10.1002/hup.1245.
40. Hegde, D.; Bhargav, P.H.; Bhargav, H.; Babu, H.; Varsha, K.A.; Raghuram, N. Feasibility and Pilot Efficacy Testing of Integrated Yoga and Shirodhara (Ayurvedic Oil-Dripping) Intervention on Clinical Symptoms, Cognitive Functions and Sleep Quality of Adults with Anxiety Disorder. *Int. J. Yoga* **2020**, *13*, 32–41. https://doi.org/10.4103/ijoy.IJOY_44_19.
41. Hoffman, J.R.; Markus, I.; Dubnov-Raz, G.; Gepner, Y. Ergogenic Effects of 8 Days of Sceletium Tortuosum Supplementation on Mood, Visual Tracking, and Reaction in Recreationally Trained Men and Women. *J. Strength Cond. Res.* **2020**, *34*, 2476–2481. https://doi.org/10.1519/JSC.0000000000003693.
42. Hughes, S.; Rondeau, M.; Shannon, S.; Sharp, J.; Ivins, G.; Lee, J.; Taylor, I.; Bendixsen, B. A Holistic Self-learning Approach for Young Adult Depression and Anxiety Compared to Medication-Based Treatment-As-Usual. *Community Ment. Health J.* **2021**, *57*, 392–402. https://doi.org/10.1007/s10597-020-00666-9.
43. Ibero-Baraibar, I.; Perez-Cornago, A.; Ramirez, M.J.; Martínez, J.A.; Zulet, M.A. An Increase in Plasma Homovanillic Acid with Cocoa Extract Consumption Is Associated with the Alleviation of Depressive Symptoms in Overweight or Obese Adults on an Energy Restricted Diet in a Randomized Controlled Trial. *J. Nutr.* **2016**, *146*, 897S–904S. https://doi.org/10.3945/jn.115.222828.
44. Inoue, T.; Kobayashi, Y.; Mori, N.; Sakagawa, M.; Xiao, J.Z.; Moritani, T.; Sakane, N.; Nagai, N. Effect of combined bifidobacteria supplementation and resistance training on cognitive function, body composition and bowel habits of healthy elderly subjects. *Benef. Microbes* **2018**, *9*, 843–853. https://doi.org/10.3920/BM2017.0193.
45. Jackson, P.A.; Forster, J.; Khan, J.; Pouchieu, C.; Dubreuil, S.; Gaudout, D.; Moras, B.; Pourtau, L.; Joffre, F.; Vaysse, C.; et al. Effects of Saffron Extract Supplementation on Mood, Well-Being, and Response to a Psychosocial Stressor in Healthy Adults: A Randomized, Double-Blind, Parallel Group, Clinical Trial. *Front. Nutr.* **2021**, *7*, 606124. https://doi.org/10.3389/fnut.2020.606124.
46. Jamilian, M.; Foroozanfard, F.; Kavossian, E.; Aghadavod, E.; Amirani, E.; Mahdavinia, M.; Mafi, A.; Asemi, Z. Carnitine and chromium co-supplementation affects mental health, hormonal, inflammatory, genetic, and oxidative stress parameters in women with polycystic ovary syndrome. *J. Psychosom. Obstet. Gynaecol.* **2019**, *10*, 1–9. https://doi.org/10.1080/0167482X.2018.1557144.
47. Jamilian, M.; Mansury, S.; Bahmani, F.; Heidar, Z.; Amirani, E.; Asemi, Z. The effects of probiotic and selenium co-supplementation on parameters of mental health, hormonal profiles, and biomarkers of inflammation and oxidative stress in women with polycystic ovary syndrome. *J. Ovarian Res.* **2018**, *11*, 80. https://doi.org/10.1186/s13048-018-0457-1.
48. Jamilian, M.; Samimi, M.; Mirhosseini, N.; Afshar Ebrahimi, F.; Aghadavod, E.; Talaee, R.; Jafarnejad, S.; Hashemi Dizaji, S.; Asemi, Z. The influences of vitamin D and omega-3 co-supplementation on clinical, metabolic and genetic parameters in women with polycystic ovary syndrome. *J. Affect. Disord.* **2018**, *238*, 32–38. https://doi.org/10.1016/j.jad.2018.05.027.
49. Kaplan, B.J.; Rucklidge, J.J.; Romijn, A.R.; Dolph, M. A randomised trial of nutrient supplements to minimise psychological stress after a natural disaster. *Psychiatry Res.* **2015**, *228*, 373–379. https://doi.org/10.1016/j.psychres.2015.05.080.

50. Kiecolt-Glaser, J.K.; Belury, M.A.; Andridge, R.; Malarkey, W.B.; Glaser, R. Omega-3 supplementation lowers inflammation and anxiety in medical students: A randomized controlled trial. *Brain Behav. Immun.* **2011**, *25*, 1725–1734. https://doi.org/10.1016/j.bbi.2011.07.229.
51. Kumar, T.; Srivastav, M.; Wahi, A.K.; Singh, H.K.; Singh, R. Randomized control, double blind cross-over study to clinically assess the Rasayana effect of a standardized extract of Brahmi (*Bacopa monniera*) in adult human volunteers. *Int. J. Pharm. Pharm. Sci.* **2011**, *3*, 263–266.
52. Kyrou, I.; Christou, A.; Panagiotakos, D.; Stefanaki, C.; Skenderi, K.; Katsana, K.; Tsigos, C. Effects of a hops (Humulus lupulus L.) dry extract supplement on self-reported depression, anxiety and stress levels in apparently healthy young adults: A randomized, placebo-controlled, double-blind, crossover pilot study. *Hormones* **2017**, *16*, 171–180. https://doi.org/10.14310/horm.2002.1738.
53. Lewis, J.E.; Tiozzo, E.; Melillo, A.B.; Leonard, S.; Chen, L.; Mendez, A.; Woolger, J.M.; Konefal, J. The effect of methylated vitamin B complex on depressive and anxiety symptoms and quality of life in adults with depression. *ISRN Psychiatry* **2013**, *2013*, 621453. https://doi.org/10.1155/2013/621453.
54. Li, H.-C.; Li, Q.-B.; Yang, X.-Q. [Analysis on the depression related factors in elderly patients after stroke and the effect of Chinese medicine on them]. *Zhongguo Zhong Xi Yi Jie He Za Zhi Chin. J. Integr. Tradit. West. Med.* **2010**, *30*, 1133–1137.
55. Lopresti, A.L.P.; Smith, S.J.M.A.; Malvi, H.M.M.D.; Kodgule, R.M.; Wane, D.; Lopresti, A.L.; Smith, S.J.; Malvi, H.; Kodgule, R. An investigation into the stress-relieving and pharmacological actions of an ashwagandha (Withania somnifera) extract: A randomized, double-blind, placebo-controlled study. *Medicine* **2019**, *98*, e17186–e17186. https://doi.org/10.1097/MD.0000000000017186.
56. Macpherson, H.; Rowsell, R.; Cox, K.H.M.; Scholey, A.; Pipingas, A. Acute mood but not cognitive improvements following administration of a single multivitamin and mineral supplement in healthy women aged 50 and above: A randomised controlled trial. *Age* **2015**, *37*, 9782. https://doi.org/10.1007/s11357-015-9782-0.
57. Mazidi, M.; Shemshian, M.; Mousavi, S.H.; Norouzy, A.; Kermani, T.; Moghiman, T.; Sadeghi, A.; Mokhber, N.; Ghayour-Mobarhan, M.; Ferns, G.A.A. A double-blind, randomized and placebo-controlled trial of Saffron (*Crocus sativus* L.) in the treatment of anxiety and depression. *J. Complement. Integr. Med.* **2016**, *13*, 195–199. https://doi.org/10.1515/jcim-2015-0043.
58. Neri, M.; Andermarcher, E.; Pradelli, J.M.; Salvioli, G. Influence of a double blind pharmacological trial on two domains of well-being in subjects with age associated memory impairment. *Arch. Gerontol. Geriatr.* **1995**, *21*, 241–252. https://doi.org/10.1016/0167-4943(95)00659-9.
59. Nakasone, Y.; Iwaraa, Y.; Yamaguchi, H. Effects of garlic-egg yolk combination diet on perceived mood disturbance and sleep difficulty in workers with mild work-stresses—Randomized, double-blind, placebo-controlled parallel-group trial. *Jpn. Pharmacol. Ther.* **2015**, *43*, 1007–1016.
60. Noah, L.; Dye, L.; Bois De Fer, B.; Mazur, A.; Pickering, G.; Pouteau, E. Effect of magnesium and vitamin B6 supplementation on mental health and quality of life in stressed healthy adults: Post-hoc analysis of a randomised controlled trial. *Stress Health J. Int. Soc. Investig. Stress* **2021**, *37*, 1000–1009. https://doi.org/10.1002/smi.3051.
61. Omori, K.; Tanaka, Y.; Kawabata, H.; Asai, K. Effects of the long-term intake of an amino acid-containing supplement on sleep, fatigue, and stress: A double-blind, crossover, placebo-controlled study. *Jpn. Pharmacol. Ther.* **2017**, *45*, 921–927.
62. Ostadmohammadi, V.; Jamilian, M.; Bahmani, F.; Asemi, Z. Vitamin D and probiotic co-supplementation affects mental health, hormonal, inflammatory and oxidative stress parameters in women with polycystic ovary syndrome. *J. Ovarian Res.* **2019**, *12*, 5. https://doi.org/10.1186/s13048-019-0480-x.
63. Pouteau, E.; Kabir-Ahmadi, M.; Noah, L.; Mazur, A.; Dye, L.; Hellhammer, J.; Pickering, G.; Dubray, C. Superiority of magnesium and vitamin B6 over magnesium alone on severe stress in healthy adults with low magnesemia: A randomized, single-blind clinical trial. *PloS ONE* **2018**, *13*, e0208454. https://doi.org/10.1371/journal.pone.0208454.
64. Raygan, F.; Ostadmohammadi, V.; Bahmani, F.; Asemi, Z. The effects of vitamin D and probiotic co-supplementation on mental health parameters and metabolic status in type 2 diabetic patients with coronary heart disease: A randomized, double-blind, placebo-controlled trial. *Prog. Neuro-Psychopharmacol. Biol. Psychiatry* **2018**, *84*, 50–55. https://doi.org/10.1016/j.pnpbp.2018.02.007.
65. Remenapp, A.; Coyle, K.; Orange, T.; Lynch, T.; Hooper, D.; Hooper, S.; Conway, K.; Hausenblas, H.A. Efficacy of Withania somnifera supplementation on adult's cognition and mood. *J. Ayurveda Integr. Med.* **2021**, *13*, 100510. https://doi.org/10.1016/j.jaim.2021.08.003.

66. Roca, M.; Kohls, E.; Gili, M.; Watkins, E.; Owens, M.; Hegerl, U.; van Grootheest, G.; Bot, M.; Cabout, M.; Brouwer, I.A.; et al. Prevention of depression through nutritional strategies in high-risk persons: Rationale and design of the MooDFOOD prevention trial. *BMC Psychiatry* **2016**, *16*, 192. https://doi.org/10.1186/s12888-016-0900-z.
67. Saccarello, A.; Montarsolo, P.; Massardo, I.; Picciotto, R.; Pedemonte, A.; Castagnaro, R.; Brasesco, P.C.; Guida, V.; Picco, P.; Fioravanti, P.; et al. Oral Administration of S-Adenosylmethionine (SAMe) and Lactobacillus Plantarum HEAL9 Improves the Mild-To-Moderate Symptoms of Depression: A Randomized, Double-Blind, Placebo-Controlled Study. *Prim. Care Companion CNS Disord.* **2020**, *22*, 23164. https://doi.org/10.4088/PCC.19m02578.
68. Salleh, R.M.; Kuan, G.; Aziz, M.N.A.; Rahim, M.R.A.; Rahayu, T.; Sulaiman, S.; Kusuma, D.W.Y.; Adikari, A.M.G.C.P.; Razam, M.S.M.; Radhakrishnan, A.K.; et al. Effects of Probiotics on Anxiety, Stress, Mood and Fitness of Badminton Players. *Nutrients* **2021**, *13*, 1783–1783. https://doi.org/10.3390/nu13061783.
69. Salve, J.; Pate, S.; Debnath, K.; Langade, D. Adaptogenic and Anxiolytic Effects of Ashwagandha Root Extract in Healthy Adults: A Double-blind, Randomized, Placebo-controlled Clinical Study. *Cureus* **2019**, *11*, e6466. https://doi.org/10.7759/cureus.6466.
70. Sanders, K.M.; Stuart, A.L.; Williamson, E.J.; Jacka, F.N.; Dodd, S.; Nicholson, G.; Berk, M. Annual high-dose vitamin D3 and mental well-being: Randomised controlled trial. *Br. J. Psychiatry J. Ment. Sci.* **2011**, *198*, 357–364. https://doi.org/10.1192/bjp.bp.110.087544.
71. Schaafsma, A.; Mallee, L.; van den Belt, M.; Floris, E.; Kortman, G.; Veldman, J.; van den Ende, D.; Kardinaal, A. The Effect of A Whey-Protein and Galacto-Oligosaccharides Based Product on Parameters of Sleep Quality, Stress, and Gut Microbiota in Apparently Healthy Adults with Moderate Sleep Disturbances: A Randomized Controlled Cross-Over Study. *Nutrients* **2021**, *13*, 2204–2204. https://doi.org/10.3390/nu13072204.
72. Scholey, A.; Benson, S.; Gibbs, A.; Perry, N.; Sarris, J.; Murray, G. Exploring the Effect of Lactium™ and Zizyphus Complex on Sleep Quality: A Double-Blind, Randomized Placebo-Controlled Trial. *Nutrients* **2017**, *9*, 154. https://doi.org/10.3390/nu9020154.
73. Shabani, A.; Foroozanfard, F.; Kavossian, E.; Aghadavod, E.; Ostadmohammadi, V.; Reiter, R.J.; Eftekhar, T.; Asemi, Z. Effects of melatonin administration on mental health parameters, metabolic and genetic profiles in women with polycystic ovary syndrome: A randomized, double-blind, placebo-controlled trial. *J. Affect. Disord.* **2019**, *250*, 51–56. https://doi.org/10.1016/j.jad.2019.02.066.
74. Shetty, S.K.; Rao, P.N.; U, S.; Raj, A.; Ks, S.; Sv, S. The effect of Brahmi (*Bacopa monnieri* (L.) Pennell) on depression, anxiety and stress during Covid-19. *Eur. J. Integr. Med.* **2021**, *48*, 101898. https://doi.org/10.1016/j.eujim.2021.101898.
75. Sim, M.; Hong, S.; Jung, S.; Kim, J.-S.; Goo, Y.-T.; Chun, W.Y.; Shin, D.-M. Vitamin C supplementation promotes mental vitality in healthy young adults: Results from a cross-sectional analysis and a randomized, double-blind, placebo-controlled trial. *Eur. J. Nutr.* **2022**, *61*, 447–459. https://doi.org/10.1007/s00394-021-02656-3.
76. Slow, S.; Florkowski, C.M.; Chambers, S.T.; Priest, P.C.; Stewart, A.W.; Jennings, L.C.; Livesey, J.H.; Camargo, C.A., Jr.; Scragg, R.; Murdoch, D.R. Effect of monthly vitamin D3 supplementation in healthy adults on adverse effects of earthquakes: Randomised controlled trial. *BMJ (Clin. Res. Ed.)* **2014**, *349*, g7260. https://doi.org/10.1136/bmj.g7260.
